# Transurethral resection of the prostate across continents: a meta-analysis evaluating quality of gold standard in the twenty-first century

**DOI:** 10.1007/s00345-024-05439-7

**Published:** 2025-01-24

**Authors:** Joao G. Porto, Ansh M. Bhatia, Abhishek Bhat, Maria Camila Suarez Arbelaez, Ruben Blachman-Braun, Khushi Shah, Ankur Malpani, Diana Lopategui, Thomas R. W. Herrmann, Robert Marcovich, Hemendra N. Shah

**Affiliations:** 1 Desai Sethi Urology Institute, Miller School of Medicine, University of Miami, 1120 NW 14th St #2107, 15th Floor, Miami, FL 33136 USA; 2 Department of Interventional Radiology, Miller School of Medicine, University of Miami, Miami, FL USA; 3 Seth GS Medical College and KEM Hospital, Mumbai, India; 4 Spitial Thurgau AG (STGAG), Frauenfeld, Switzerland

**Keywords:** Transurethral resection of the prostate, Benign prostatic hyperplasia, Enlarged prostate, Lower urinary tract symptoms

## Abstract

**Purpose:**

To compare outcomes of transurethral resection of the prostate (TURP) across different regions worldwide over the past two decades.

**Methods:**

A systematic review and meta-analysis of randomized clinical trials indexed to PubMed that assessed TURP. A total of 102 studies with 8,454 patients were included and grouped by continents: Europe, Asia, Africa, and Others (North America, South America, and Australia). International Prostate Symptom Score (IPSS), peak flow (Qmax), postvoid residual urine (PVR), PSA levels, prostate volume, and Sexual Health Inventory for Men scores (at 3, 12, and 36 months) were assessed, along with postoperative complications. Heterogeneity across studies was classified as low (I^2^ < 25%), moderate (I^2^ = 25–75%), or high (I^2^ > 75%).

**Results:**

TURP consistently exhibited significant enhancements in IPSS, Qmax, and PVR across various regions. Notably, PVR demonstrated high heterogeneity (I²=100%). TURP presented low complication rates with TURP syndrome (2%), bleeding (8%), and blood transfusion (6%). However, significant heterogeneity was observed, particularly for clot evacuation (I^2^ = 87%), irritative symptoms (I^2^ = 96%), and incontinence (I^2^ = 84%). The retreatment rates at 1 and 3 years were 5% and 7%, respectively, with significant differences across regions.

**Conclusion:**

Global outcomes of TURP lack a discernible trend. The substantial heterogeneity observed among continents suggests a lack of standardization. Nevertheless, uniform symptomatic improvements among patients still support TURP as the gold-standard surgical treatment for benign prostatic hyperplasia, despite variations in its results worldwide.

**Supplementary Information:**

The online version contains supplementary material available at 10.1007/s00345-024-05439-7.

## Introduction

Benign prostatic hyperplasia (BPH) stands as the primary contributor to urinary obstruction in males, and transurethral resection of the prostate (TURP) has been the durable keystone of its surgical treatment since its inception in 1926, having served as the sole minimally invasive alternative to open prostatectomy for managing enlarged prostates for an extended period [1]. Over time, it earned recognition as the “gold standard” in surgical treatment, as accumulating evidence consistently demonstrated its superior success in delivering optimal outcomes compared to other surgical options. After nearly a century, every new surgical approach for treating BPH is still benchmarked against TURP and with the rising interest in minimally invasive surgical therapies (MIST) and enucleation procedures, their utilization and comparisons to TURP have also seen a surge [2]. However, the volume of TURP-related publications has consistently increased over time, affirming its enduring role as the predominant procedure conducted globally. This underscores its continued effectiveness as a therapeutic approach and its position as a standard against which other treatments are evaluated [3, 4].

Nevertheless, the application of TURP has displayed varying patterns across distinct geographic regions, with certain areas experiencing an upsurge in these interventions, while others have noted a decrease [5–9]. Regrettably, the effectiveness of the gold standard treatment is contingent upon the surgeon's expertise, with experienced urologists demonstrating the ability to resect up to four times the amount of tissue per unit of time compared to their less seasoned counterparts [10]. Furthermore, past research has highlighted a positive relationship between high-volume urologists and better treatment results in patients undergoing TURP [11]. As time has passed, TURP has undergone technical enhancements and developments in endoscopic tools and cameras. Certain focused studies from single institutions have emphasized the positive impact of these technical improvements in reducing TURP-associated complications [12, 13]. Conversely, some authors were unable to replicate identical results [14].

Given the emergence of new techniques and the shifting trends surrounding TURP on a global scale, we posited that the quality of care delivered by TURP practitioners, and the occurrence of complications vary significantly among different geographical regions. In response to these queries, we undertook a comprehensive systematic review and meta-analysis of randomized clinical trials (RCTs) conducted across various parts of the world.

## Aims

We aimed to conduct a comprehensive assessment of the effectiveness and safety of this long-standing treatment and to identify potential trends that can inform best practices concerning TURP.

## Evidence acquisition

### Search strategy

In March 2023, we performed a PubMed query using the following search terms: “transurethral resection of the prostate,” “benign prostatic hyperplasia,” “enlarged prostate,” and “randomized clinical trial.” The search spanned from January 1st, 2000, to December 31st, 2022. Our inclusion criteria encompassed only RCTs that directly compared TURP to alternative treatments. Systematic reviews, meta-analyses, duplicated and irrelevant articles, case reports, expert opinions, and commentaries were excluded from consideration.

### Study selection

The study selection process can be seen in Supplementary Fig. 1. Initial screening of article titles and abstracts was independently performed by two authors, AB and MCS. Any discrepancies between authors were resolved through discussion and consensus. Full-text reviews were carried out on the selected articles, resulting in the inclusion of 102 eligible studies following thorough evaluation [15–116]. Data extraction was conducted independently by both AB and MCS, with cross-verification of their findings. This study was registered in the PROSPERO database (CRD42023401743) and adhered to the PRISMA guidelines for systematic reviews. This study represents the regional analysis of this registration. The selected articles were categorized based on the continents of their publication: Europe, Asia, Africa, and Others (encompassing North America, South America, and Australia). The consolidation of these four continents was necessitated by the limited number of studies available from these regions. Notably, publications originating from Turkey and Russia were included in the European group.

### Data extraction

We gathered data pertaining to various parameters, such as International Prostate Symptom Score (IPSS), maximum urine flow rate (Qmax), postvoid residual volume (PVR), prostate-specific antigen (PSA), prostate volume, Sexual Health Inventory For Men (SHIM), as well as preoperative complications like transurethral resection syndrome, bleeding, blood transfusion, clot evacuation, urinary retention, urinary tract infection (UTI), irritative symptoms, urinary incontinence, erectile dysfunction (ED), retrograde ejaculation (RE), urethral stricture (US), and bladder neck stenosis (BNS). Additionally, we assessed the incidental rate of prostate cancer (iPCa) and the need for retreatment at 1 and 3 years following TURP. Perioperative outcomes were meticulously recorded and organized in a tabular format, with data categorized at specific time points: baseline, 3 months, 12 months, and ≥ 36 months.

### Data analysis and synthesis

The mean difference (MD) in changes from baseline at each timepoint served as the outcome measure for continuous variables and was compared across all studies and subgroups based on continents and time intervals. Heterogeneity was evaluated using the I2 statistic, where < 25% denoted low heterogeneity, 25–75% indicated moderate heterogeneity, and > 75% signified high heterogeneity. Subgroup heterogeneity was quantified with the Qm statistic, with a significance level set at *p* < 0.05. Additionally, Tau2 and Cochrane’s Q were computed for subgroups and individual studies, and these results were presented using forest plots.

For complications, we calculated proportions with 95% confidence intervals, examining heterogeneity for each complication individually when stratified by continents and time. The pooled proportion estimate was determined using a random-effect model, and forest plots were employed to visualize the outcomes of this analysis. Statistical analysis was conducted in RStudio (RStudio Inc, MA, USA) using the “metaphor” package.

## Results and discussion

### Selected studies

We identified 102 studies comprising 8454 patients, with their origins as follows: Europe (55 studies), Asia (31 studies), Africa (7 studies), and Others (9 studies, including North America-2, South America-3, and Australia-4).

### Voiding outcomes: IPSS, Qmax, and PVR

**Fig. 1:**
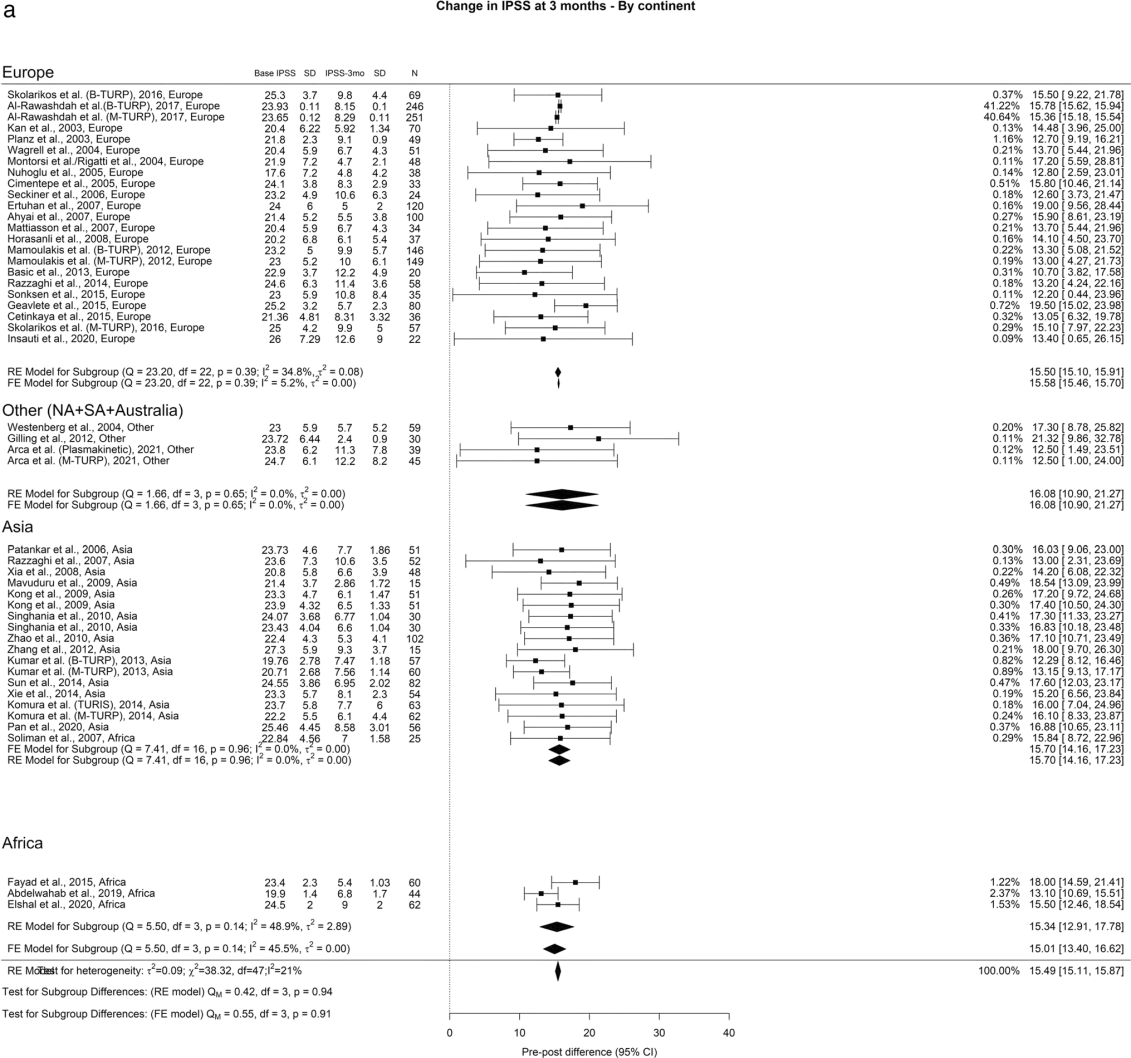

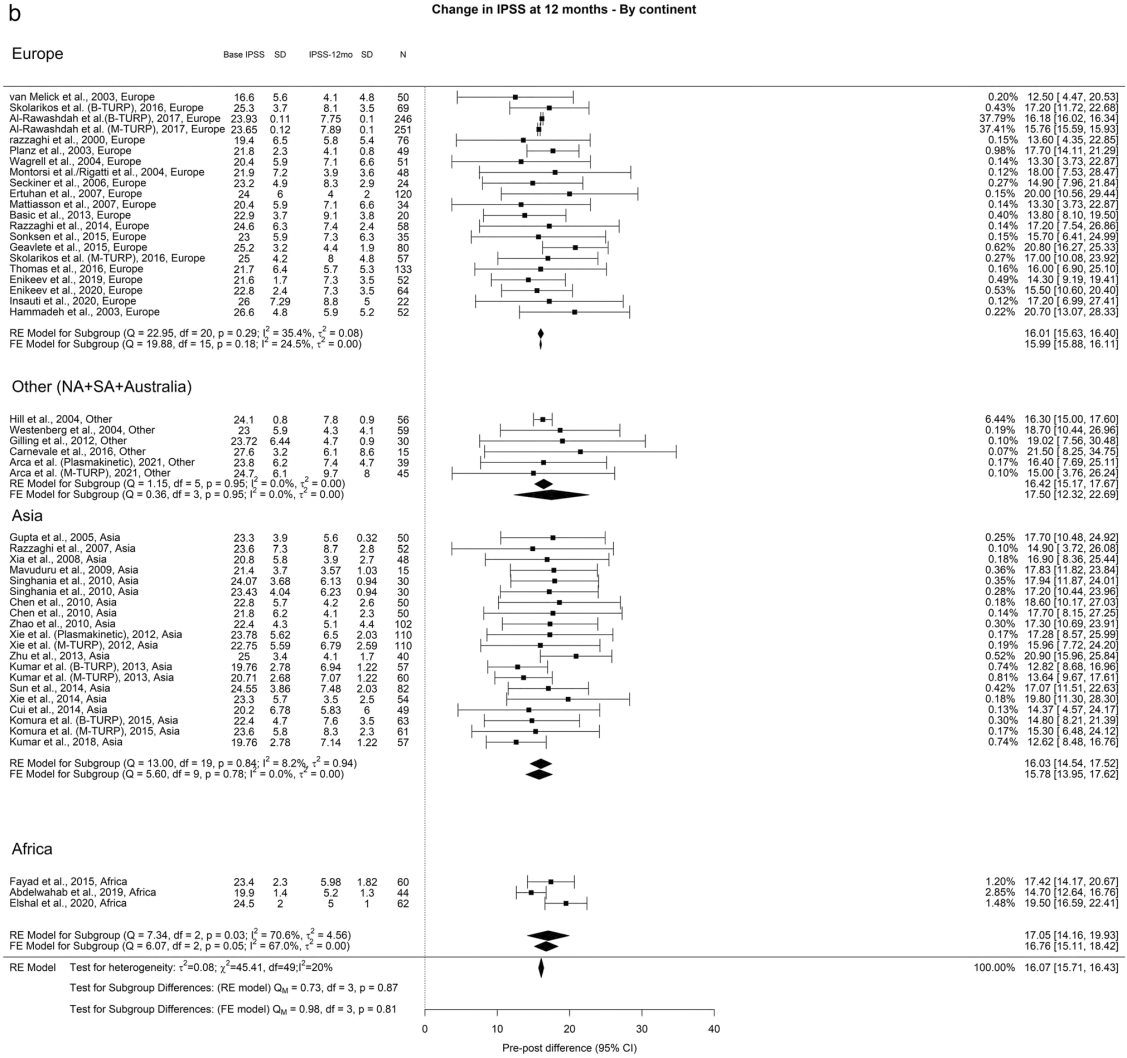

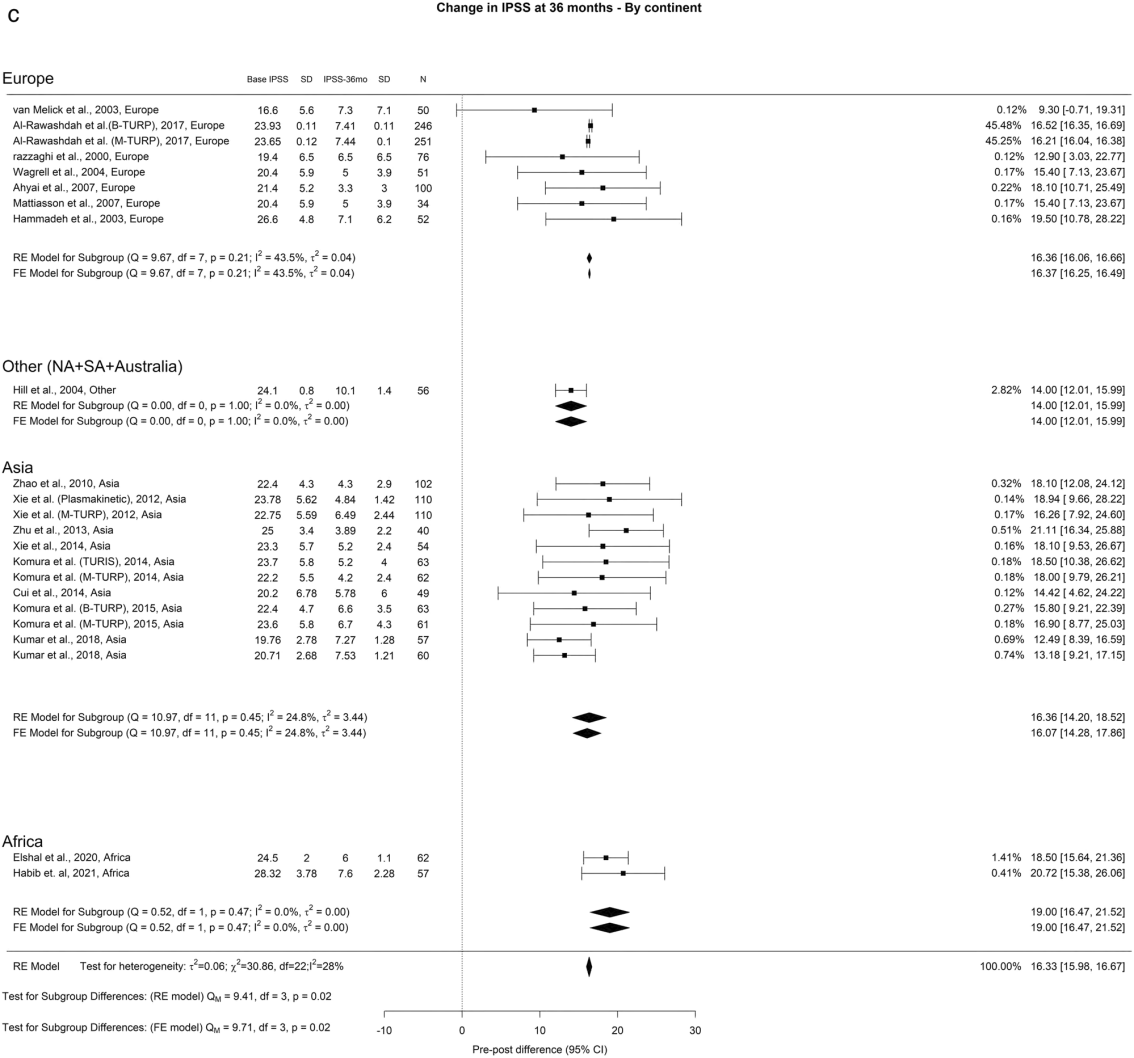
Change in IPSS by continent. **A**. At 3 months; **B**. At 12 months; **C**. At 36 months

The average reduction in IPSS over different time intervals—3 months, 12 months, and ≥ 36 months—stood at 15.4, 16, and 16.3 points, respectively. Notably, we observed a significant variance in IPSS after a 3-year follow-up (Qm = 9.41, *p* = 0.02) (Fig. 1). When we examined different regions, Europe, Other regions, Asia, and Africa showed reductions of 16.36, 14, 16.36, and 19.30 points in IPSS at 3 months, respectively.

**Fig. 2:**
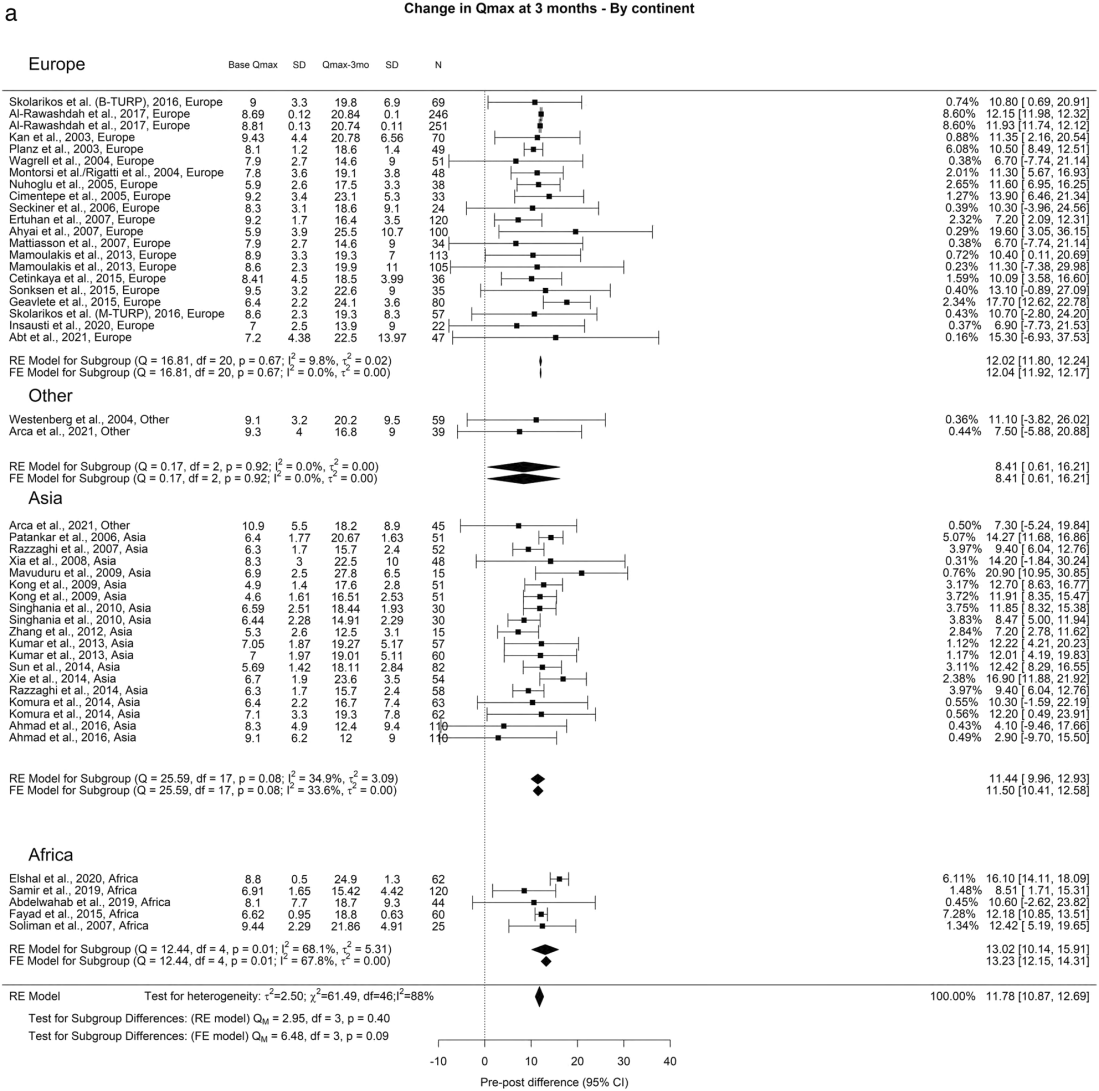

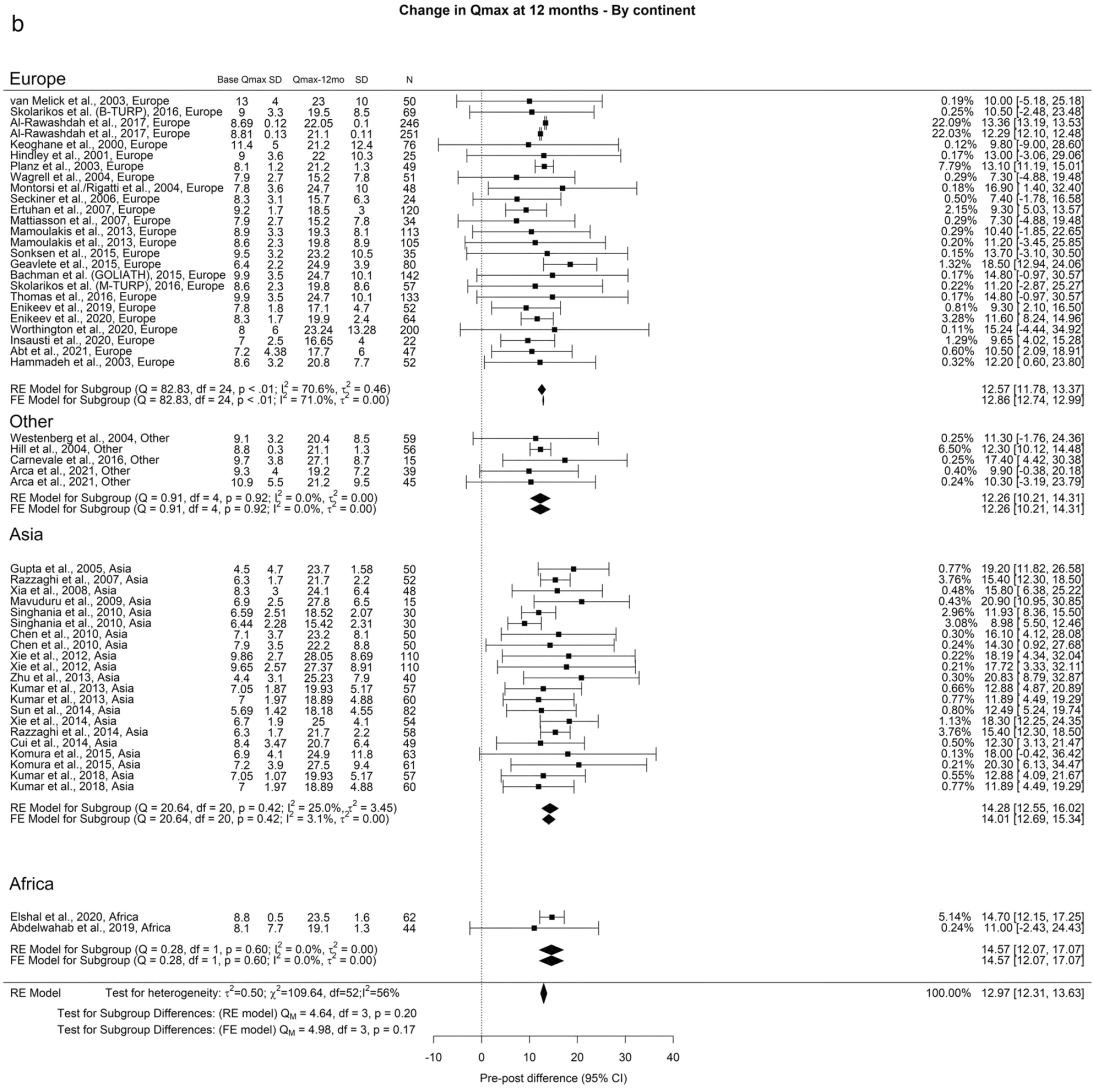

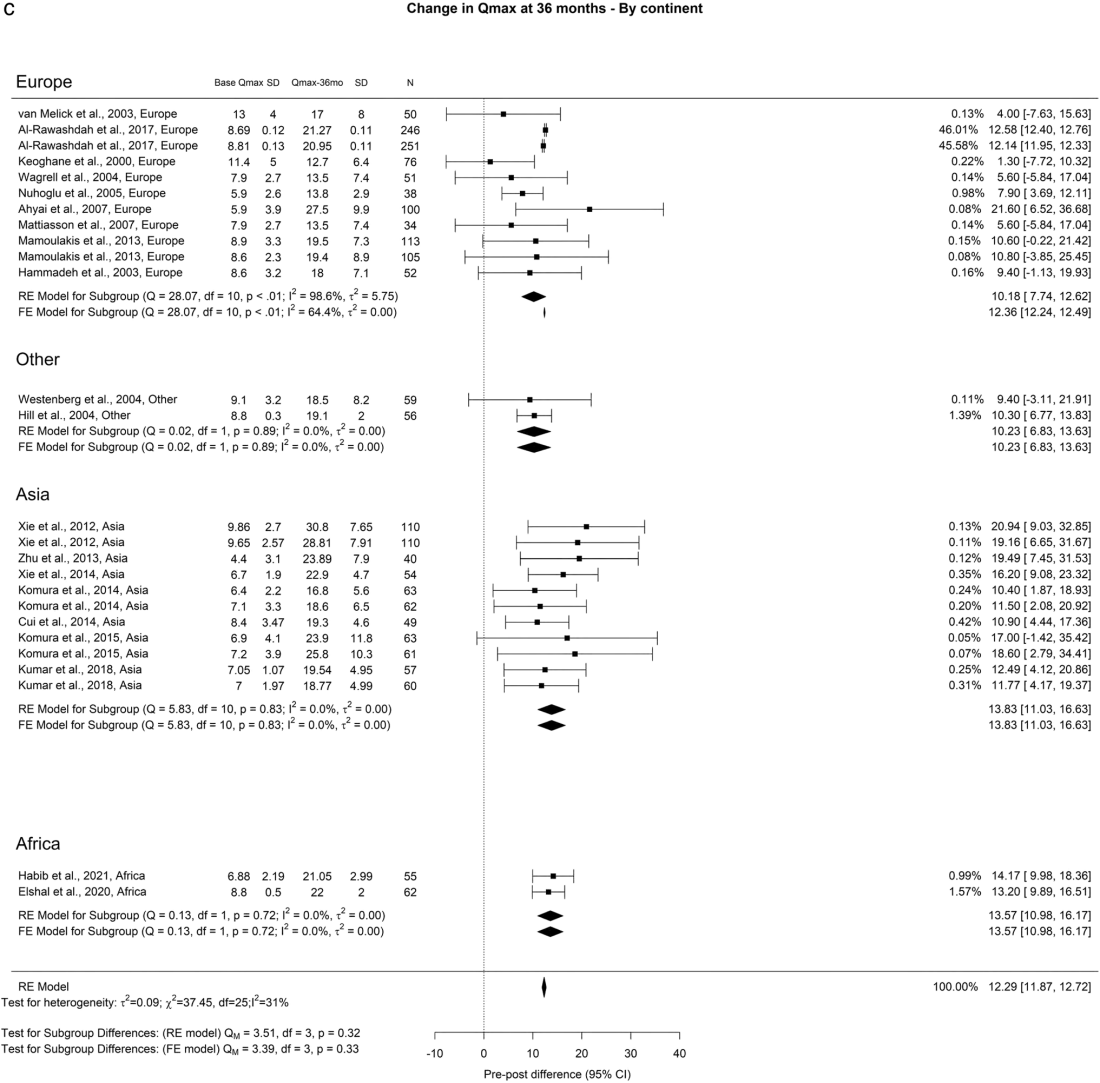
Change in Qmax by continent. **A**. At 3 months; **B**. At 12 months; **C**. At 36 months

Regarding Qmax, the overall improvements at the 3-month, 12-month, and ≥ 36-month points were 11.77 ml/s, 12.97 ml/s, and 12.29 ml/s, respectively. In our analysis we did not find any significant differences (Fig. 2).

**Fig. 3:**
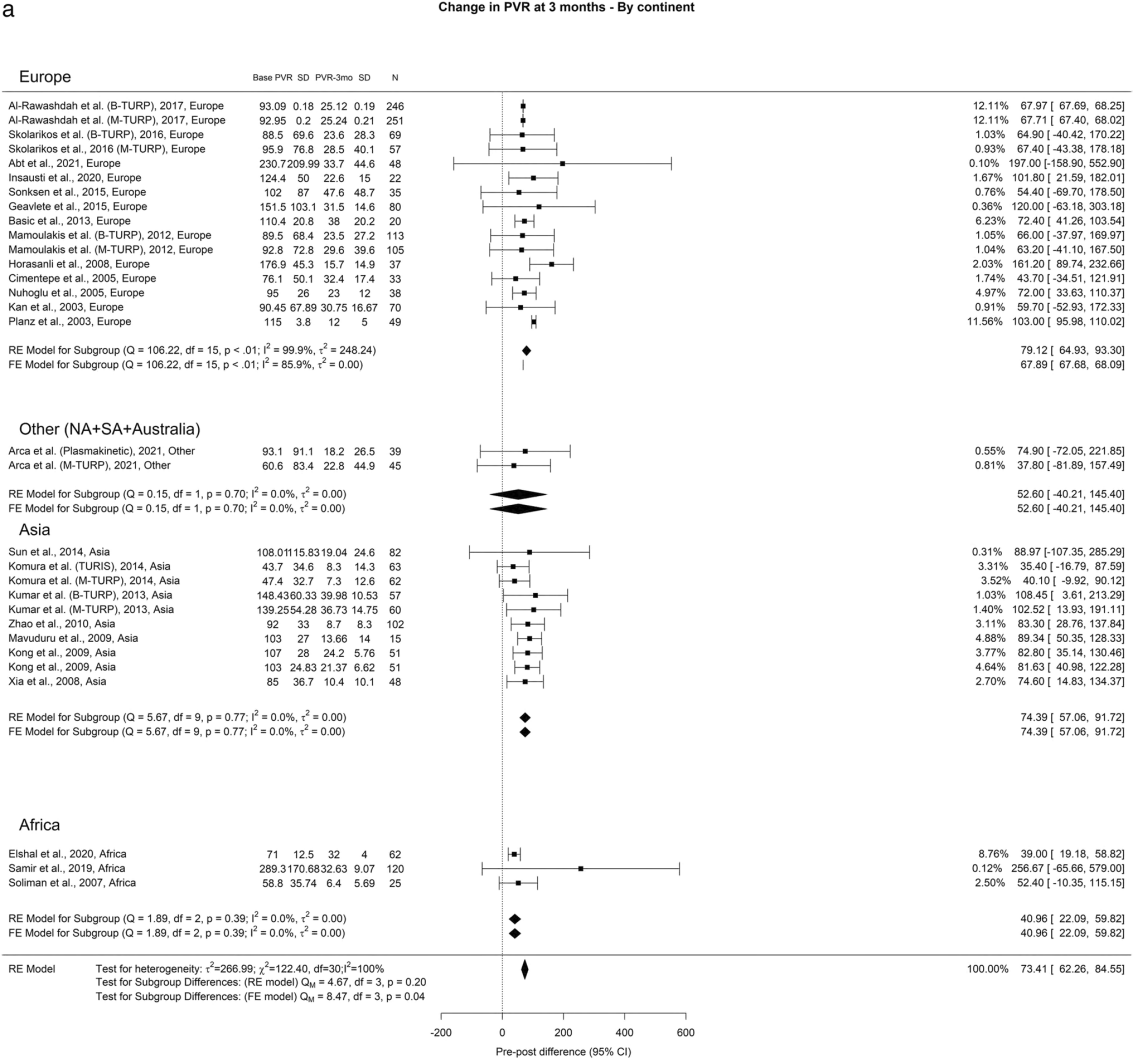

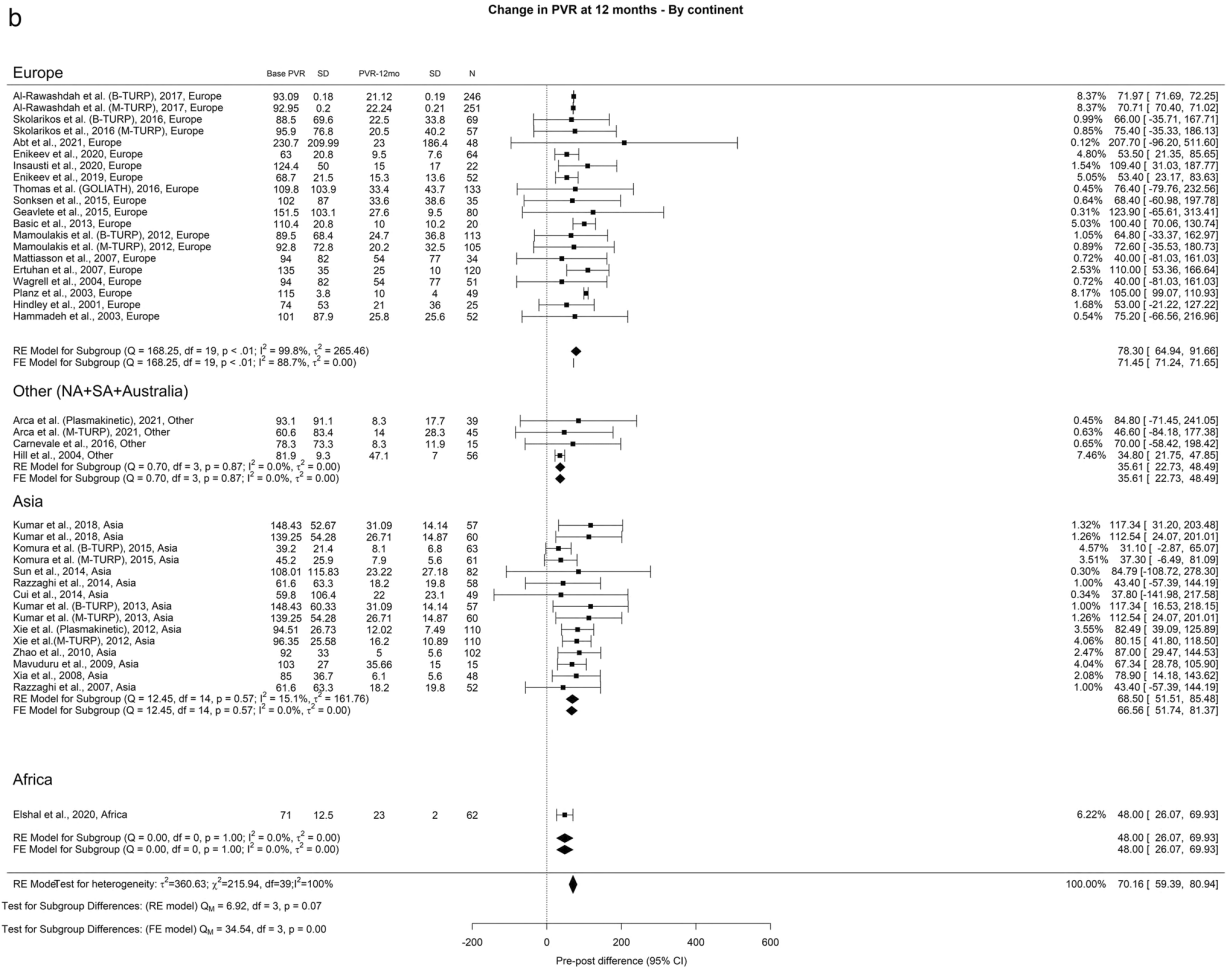

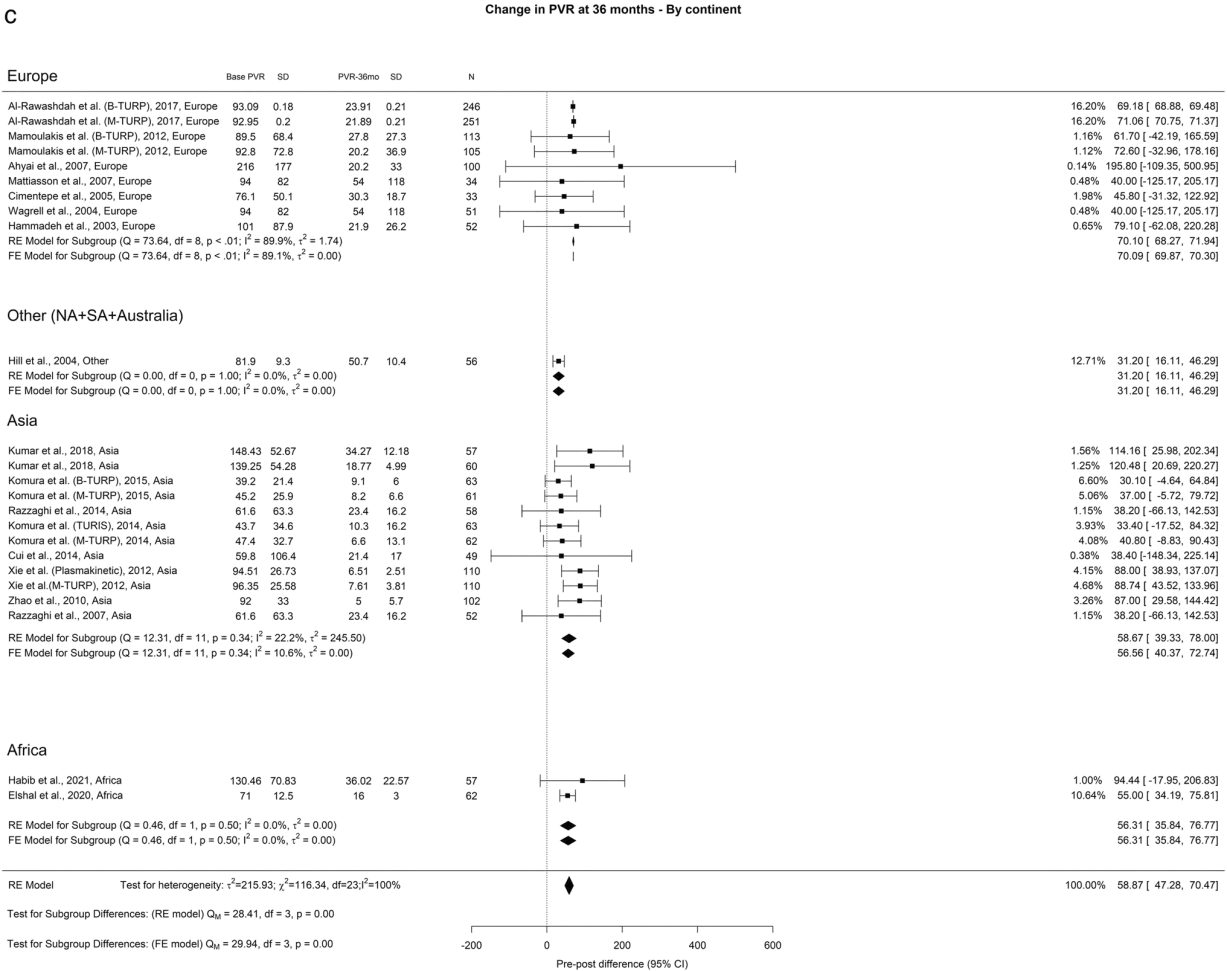
Change in PVR by continent. **A**. At 3 months; **B**. At 12 months; **C**. At 36 months

We observed an overall reduction in PVR at 3-, 12-, and ≥ 36-months of 71.73 ml, 72.94 ml, and 69.78 ml, respectively. Importantly, OH remained high (I^2^ = 100%) across all analytical perspectives. A notable discrepancy between the groups emerged at the 3-year mark, where Europe, Other regions, Asia, and Africa displayed PVR values of 78.41 ml, 35.61 ml, 68.51 ml, and 48 ml, respectively (Fig. 3).

It is worth highlighting that previous systematic reviews and meta-analyses comparing monopolar and bipolar TURP have not shown any disparities in outcomes within a 12-month timeframe [117–121]. However, studies assessing the long-term durability of these two technologies are lacking. Additionally, as noted in our research, the substantial overall heterogeneity (OH) observed in estimating PVR can be attributed to variations in the methods employed for assessing this parameter, such as bladder ultrasound or bladder scan results of both being operator dependent. These discrepancies do not appear to have clinical significance despite the statistically significant heterogeneity.

While an elevated PVR measurement can potentially indicate treatment inadequacy and guide decisions on appropriate interventions, there exists no universally accepted threshold for defining a concerning PVR [122].

### Sexual outcomes: SHIM, erectile dysfunction, and retrograde ejaculation

**Fig. 4:**
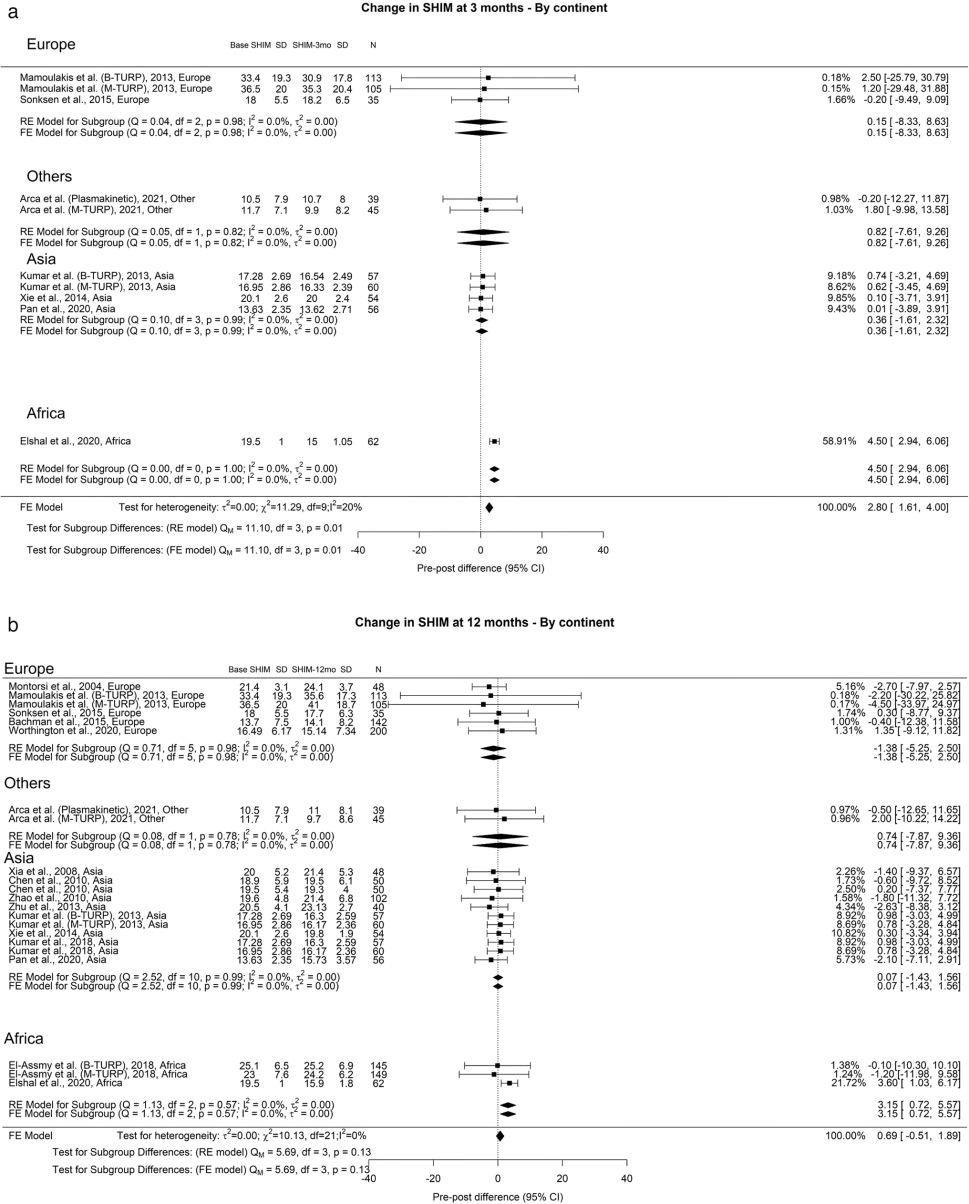

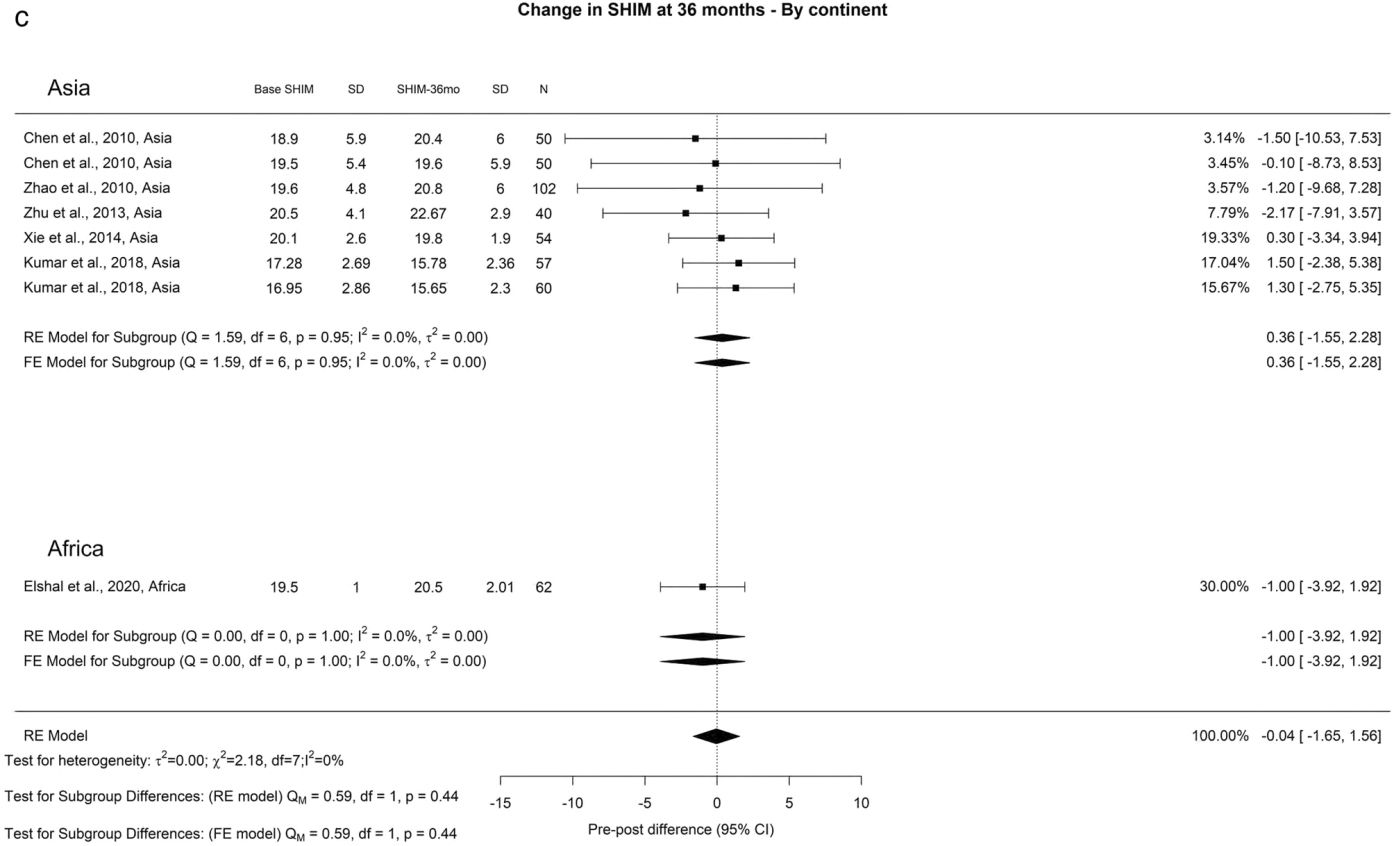
Change in SHIM by continent. **A**. At 3 months; **B**. At 12 months; **C**. At 36 months

**Fig. 5:**
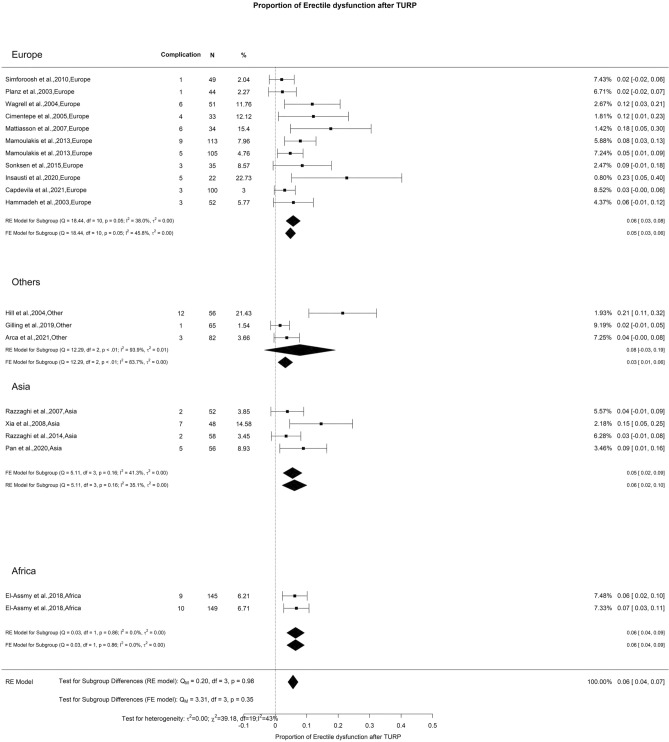
Proportion of Erectile Dysfunction after TURP

**Fig. 6:**
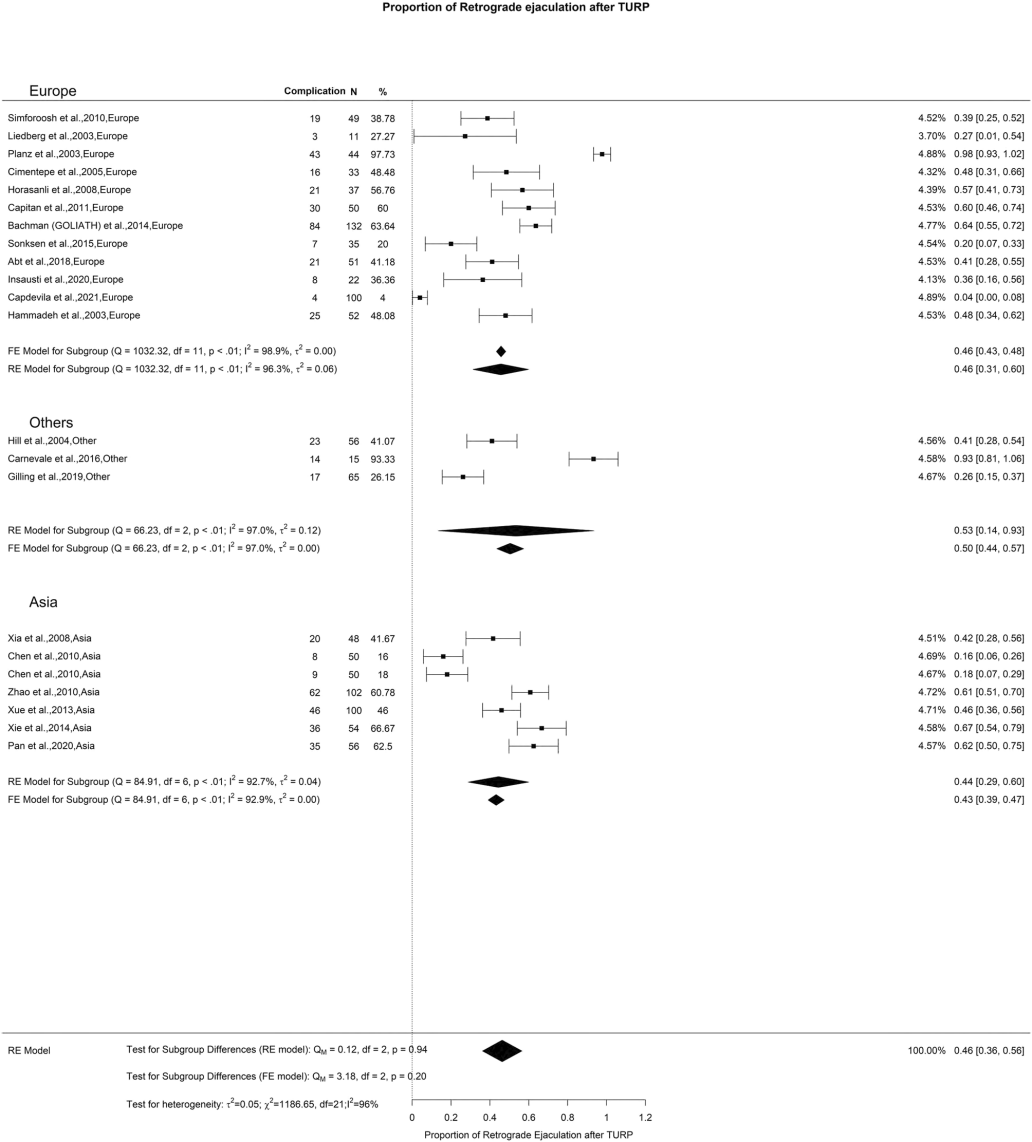
Proportion of Retrograde Ejaculation after TURP

The SHIM scores exhibited a general downward trend at the 3-and 12-months of 2.80 and 0.69 points, respectively. At follow-ups ≥ 36 months, there was an overall increase in SHIM scores of 0.4 points. We observed a noteworthy difference at the 3-month interval (Qm = 11.1, *p* = 0.01). In this context, Europe, Others, Asia, and Africa displayed SHIM score changes of 0.15, 0.82, 0.36, and 4.5, respectively (Fig. 4). Moreover, the overall reported incidence of ED following TURP was 6%, characterized by moderate OH (I^2^ = 43%) (Fig. 5). Regarding RE, the overall reported incidence was 46%, accompanied by high OH (I^2^ = 96%) (Fig. 6). We did not identify any significant differences in the incidence of ED and RE following TURP.

Our findings are consistent with numerous research studies that indicate TURP does not have a detrimental effect on erectile function and may even result in improvement [123–127]. Any minor deterioration observed beyond a 3-year follow-up period could potentially be attributed to age-related declines in sexual function [128]. RE following TURP is reported to occur in 50–70% of cases [129]. While the literature does mention technical modifications aimed at preserving peri-montanal tissue to maintain antegrade ejaculation, we did not come across any RCTs that analyzed these specific technical alterations [130, 131].

### Early postoperative complications—TURP syndrome

**Fig. 7:**
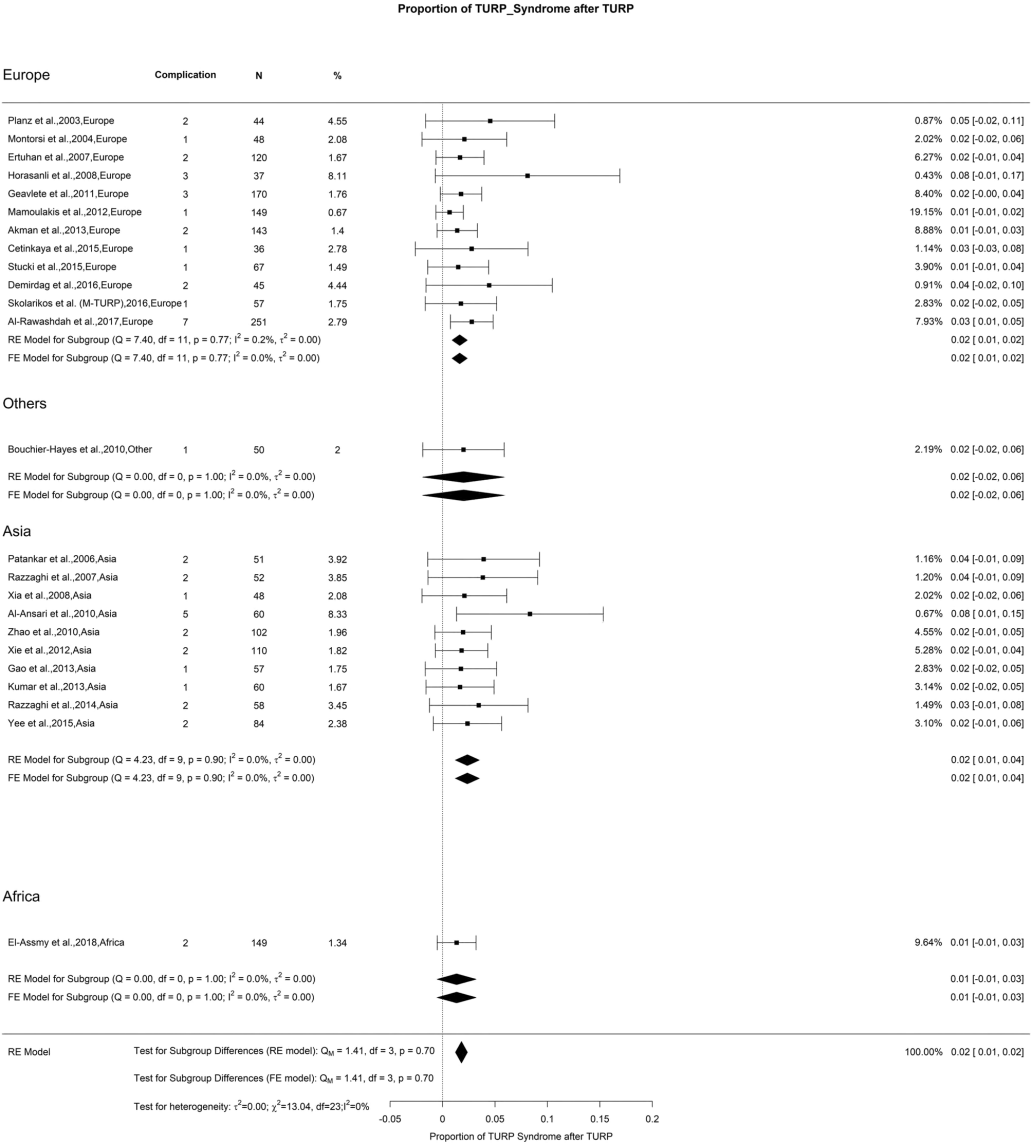
Proportion of TURP syndrome after TURP

The overall rate of TURP syndrome was 2%. We found no significant difference in the risk of this syndrome with low OH. Other authors also noted that TURP syndrome has an incidence of 0.5–8%, with a declining trend [132]. The adoption of bipolar TURP, which uses normal saline as an irrigation fluid, seems to reduce this severe and potentially life-threatening complication associated with TURP [133] (Fig. 7).

### Early postoperative complications—bleeding, blood transfusion, and clot evacuation

**Fig. 8:**
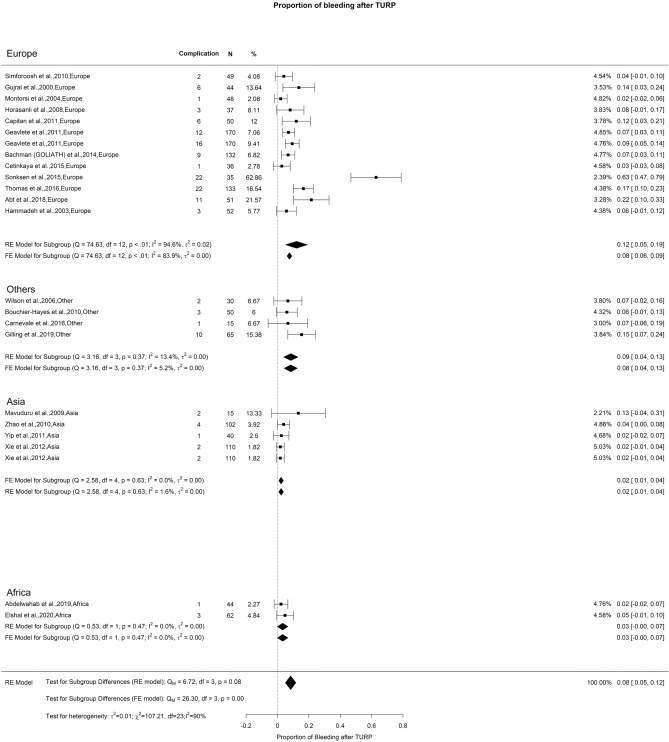
Proportion of Bleeding after TURP

**Fig. 9:**
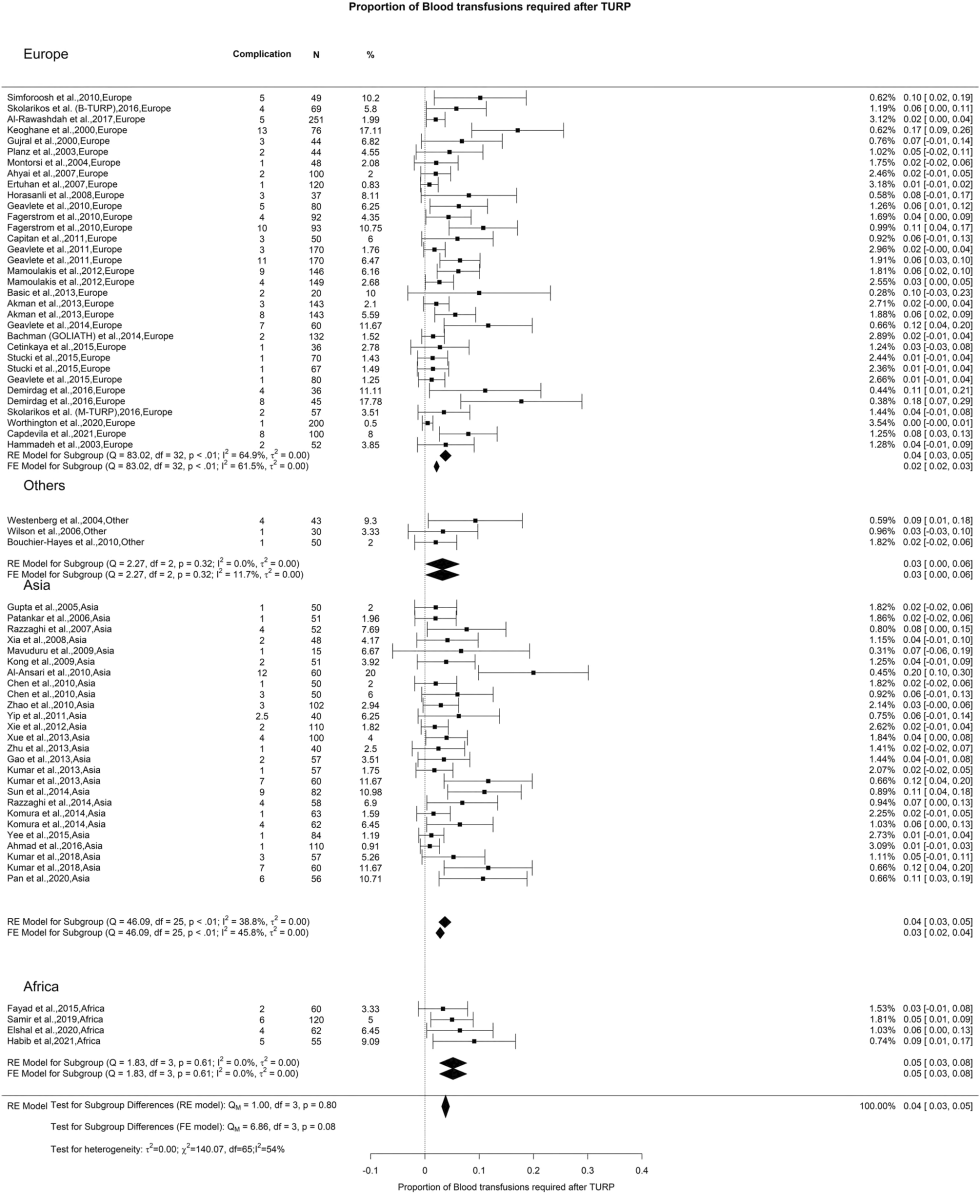
Proportion of Blood Transfusion after TURP

**Fig. 10:**
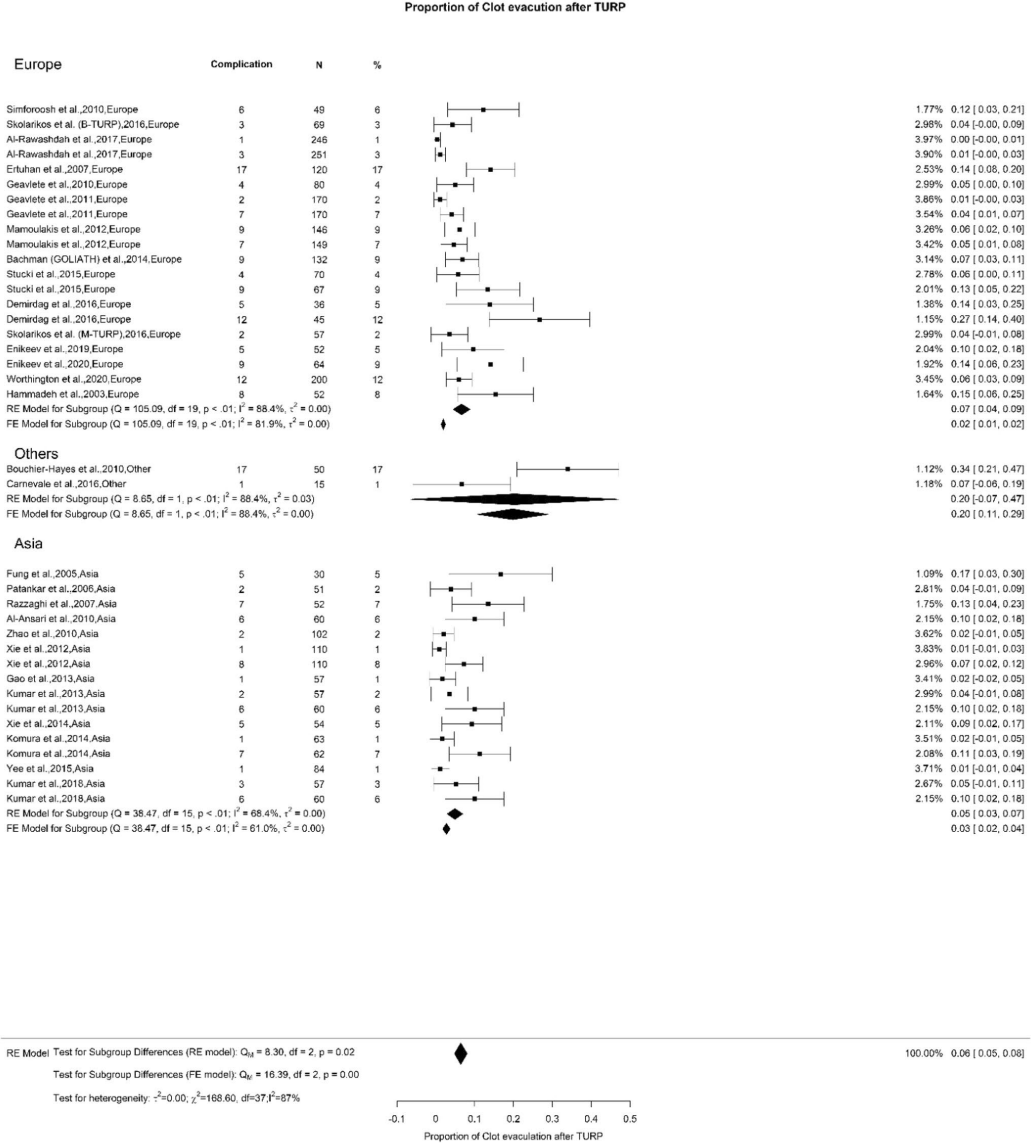
Proportion of Clot Evacuation after TURP

We observed an overall incidence of post-TURP bleeding and blood transfusion of 8% and 6%, respectively. We did not observe any difference when analyzing these variables (Figs. 8 and 9). However, there was a notable variation in the incidence of clot evacuation. While the overall rate for this complication was 6%, we found in Europe, Others, and Asia a rate of 6%, 20%, and 5%, respectively (Qm = 7.95, *p* = 0.02) (Fig. 10).

The primary factor contributing to the elevated OH in reported bleeding incidence stemmed from discrepancies in including intraoperative and/or postoperative hematuria when documenting bleeding outcomes. The studies exhibited a deficiency in providing a precise and replicable definition for quantifying bleeding objectively. Given that the criteria for blood transfusion after TURP are typically standardized, there were no noteworthy variations in its occurrence.

### Early postoperative complications: urinary retention

**Fig. 11:**
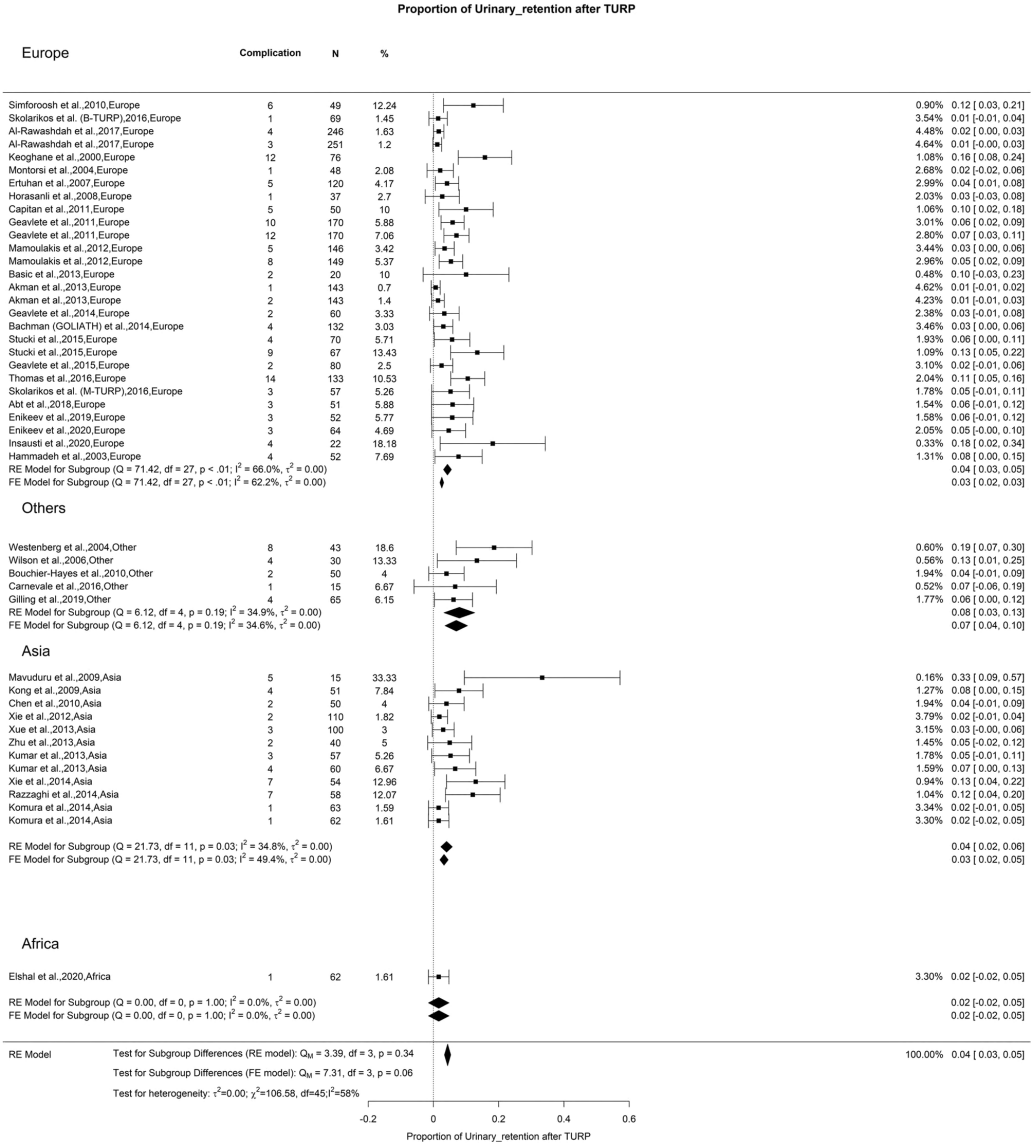
Proportion of Urinary Retention after TURP

The general incidence of urinary retention following TURP stood at 4% among individuals with moderate OH (I^2^ = 54%) (Fig. 11). Our analysis did not reveal any significant differences. Comparable findings were reported at 5.8% in a comprehensive study by Reich and colleagues, which included 10,654 patients. These researchers observed that patients who were catheterized before surgery had the highest risk of experiencing urinary retention after undergoing TURP [134].

### Early postoperative complications: urinary tract infection

**Fig. 12:**
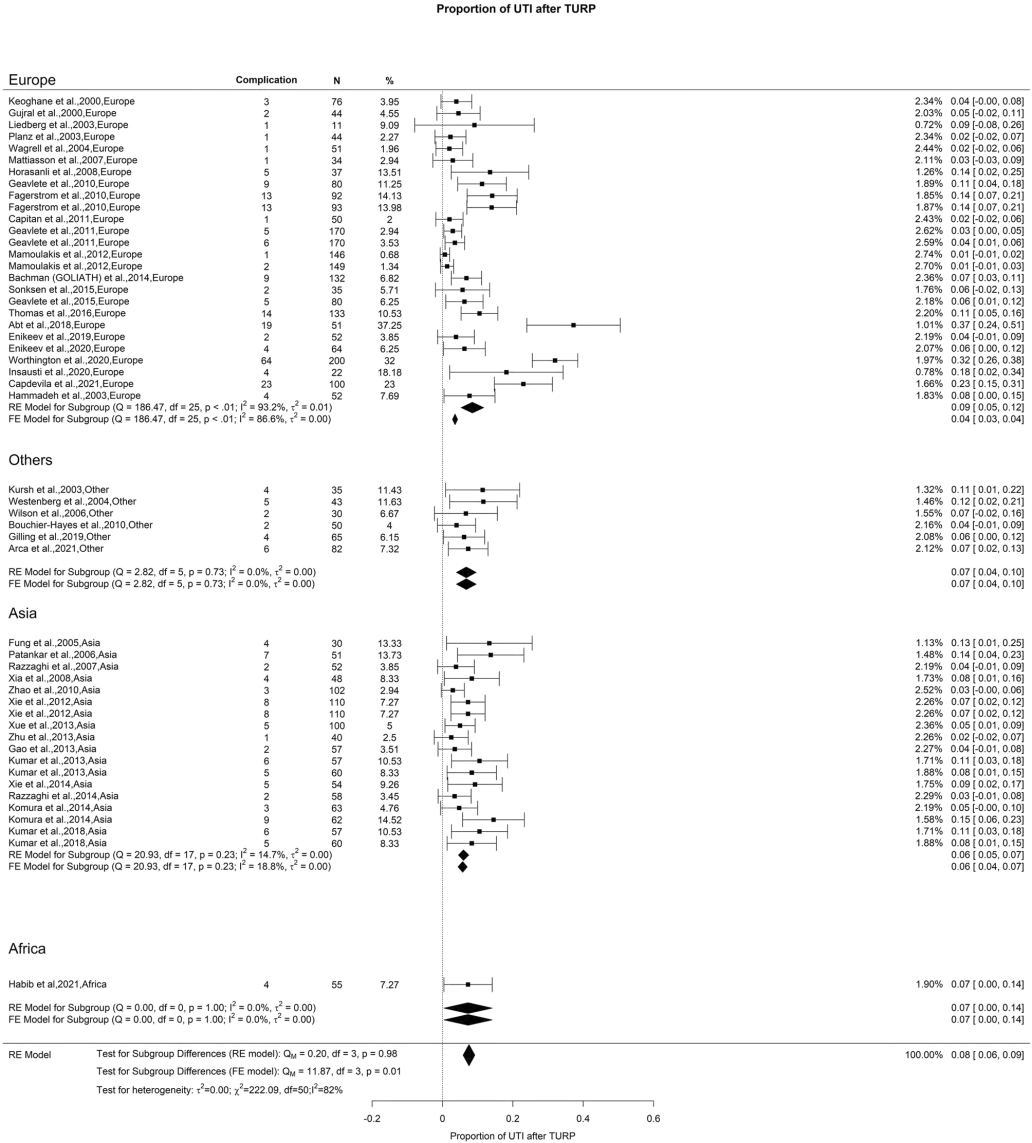
Proportion of Urinary Tract Infection after TURP

We observed a total UTI incidence of 8% in cases with high OH (I^2^ = 81%). Our examination did not uncover any notable distinctions among studies conducted on different continents (Fig. 12).

It is important to emphasize that in many studies, there is a lack of uniform and precise definition regarding this complication. There is often unclear information about whether the UTIs were symptomatic or asymptomatic and whether the diagnosis relied solely on clinical symptoms or was confirmed through a positive urine culture. A multinational study underscored the moderate adherence to infection control guidelines, with preoperative urine cultures collected for only 59% of patients [135]. Postoperative UTI remains a significant concern, as it represents one of the most frequent reasons for hospital readmission within the 30 days following TURP [136].

### Early postoperative complications: irritative symptoms

**Fig. 13:**
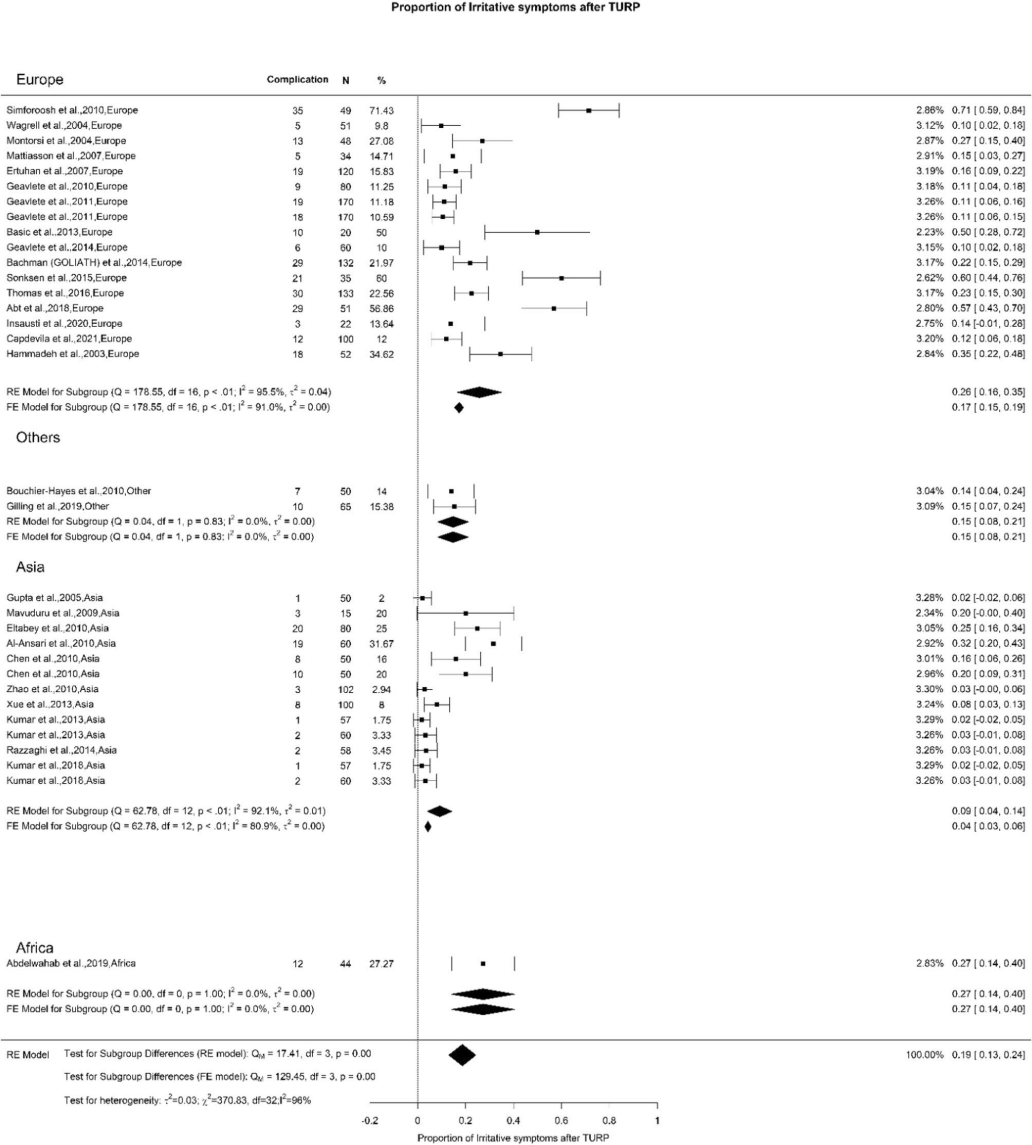
Proportion of Irritative Symptoms after TURP

We noted an overall rate of irritative symptoms after TURP of 19% with high OH (I^2^ = 96%). We found a significant difference, in which Europe, Others, Asia, and Africa demonstrated 26%, 15%, 9%, and 27%, respectively (Qm = 12.69, *p* = 0.01) (Fig. 13). This difference can be explained by variability in reporting this outcome.

### Early postoperative complications: urinary incontinence

**Fig. 14:**
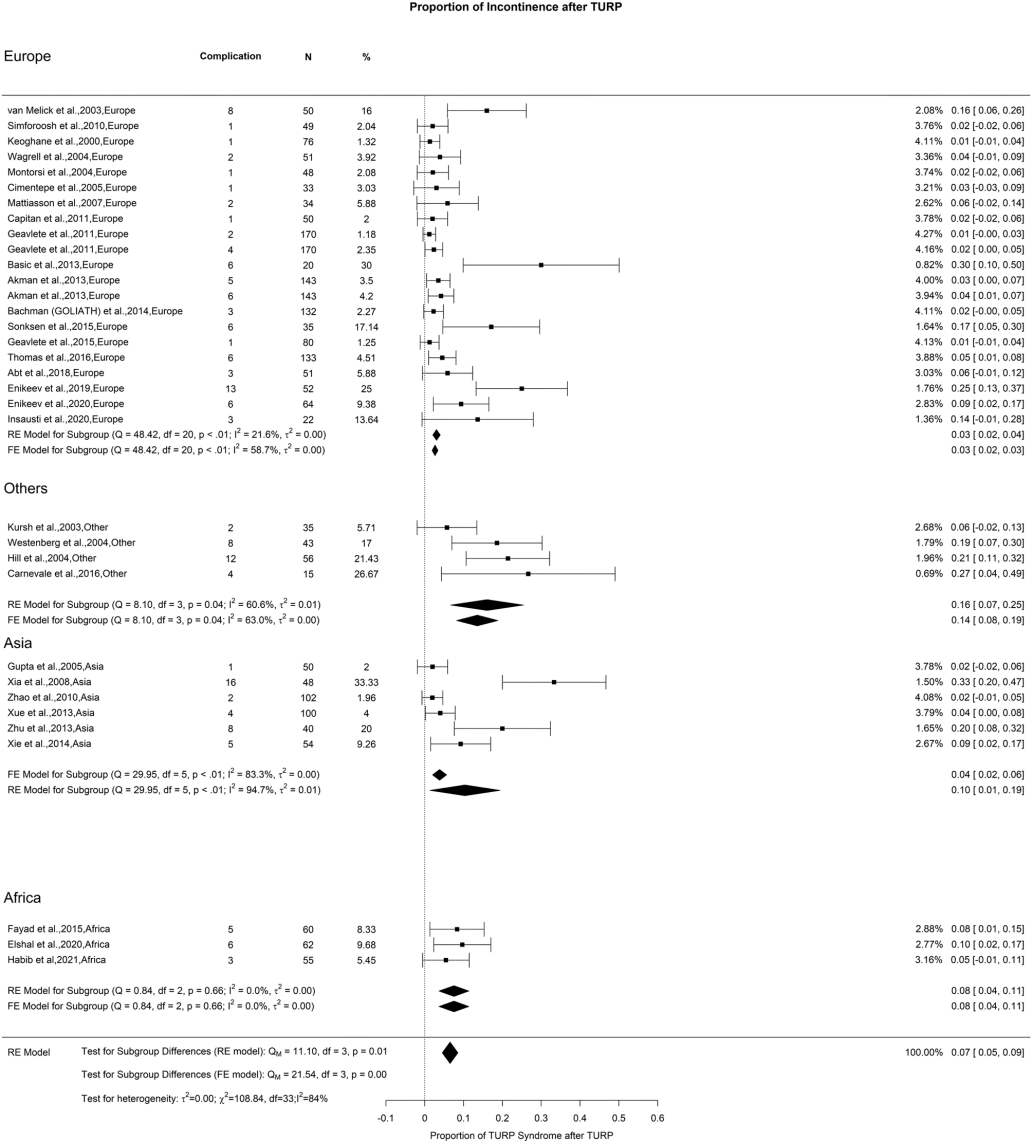
Proportion of Urinary Incontinence after TURP

The overall rate of urinary incontinence reported in all studies included was 7%, with high OH (I^2^ = 84%). A significant difference was found, in which Europe, Others, Asia, and Africa demonstrated 3%, 16%, 4%, and 8%, respectively (Qm = 11.1, *p* = 0.01) (Fig. 14).

In the present study, we did not conduct separate analyses of the sub-types of urinary incontinence or specify the postoperative follow-up period for reporting incontinence due to the considerable variability observed in different studies. However, it is crucial to acknowledge that urinary incontinence following BPH procedures significantly impacts patients' quality of life, with age being a significant risk factor [137, 138]. To advance the understanding of postoperative urinary incontinence in the context of various BPH surgeries, it is essential to establish a standardized reporting approach in all relevant studies. Although the literature suggests a comparable incontinence rate across all treatment modalities for enlarged prostate, there is a consistent trend of improvement in the postoperative period over time [139].

### Late postoperative complications: urethral stricture

**Fig. 15:**
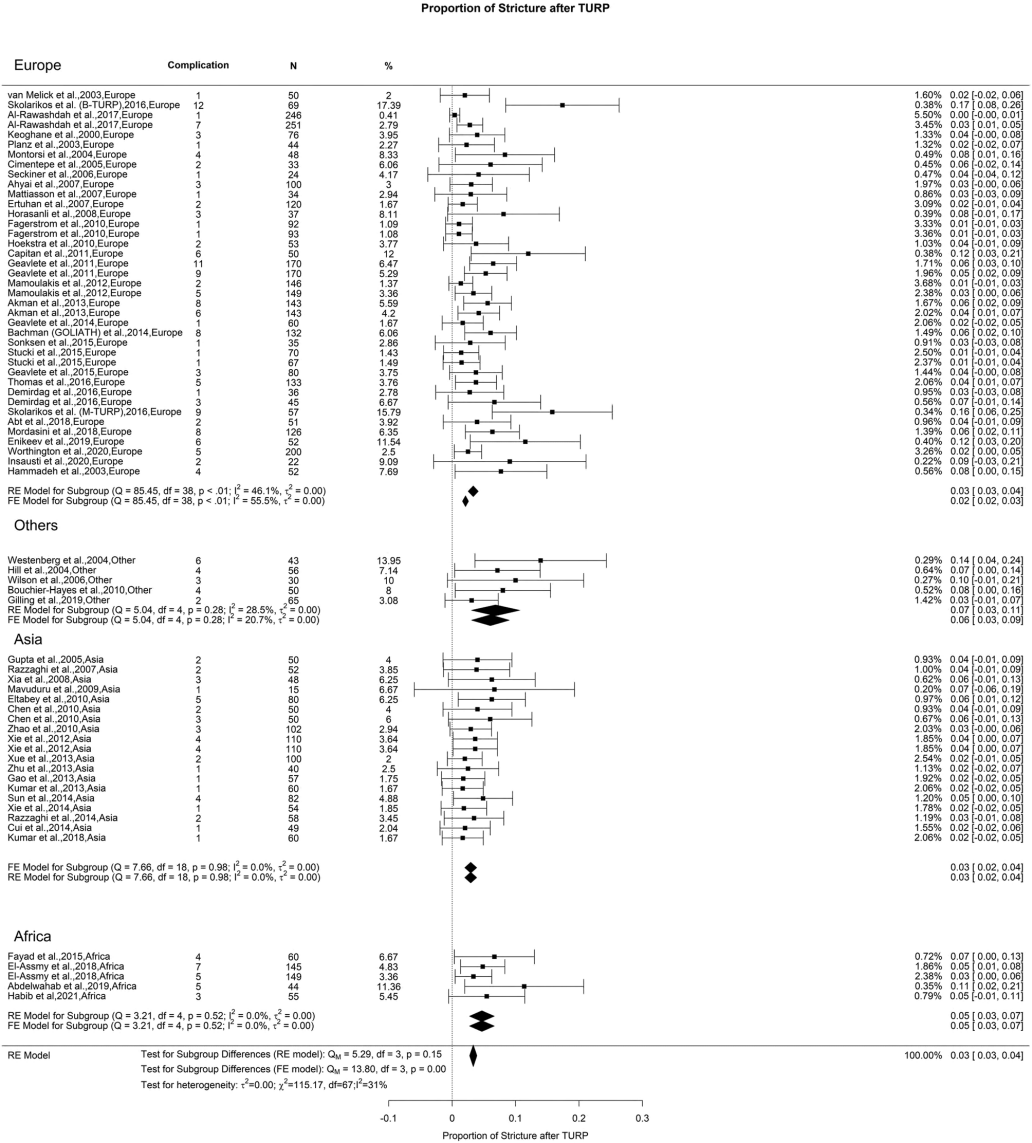
Proportion of Urethral Stricture after TURP

In this study, the US rate after TURP was 3%, accompanied by a moderate OH (I^2^ = 32%). Our analysis did not show a significant difference (Fig. 15). Past literature indicates that the US rate post-TURP varies between 2.2 and 9.8% [133]. Pirola et al. 's research showed that TURP demonstrates the highest US rate when contrasted with enucleation and ablation methods [140]. Factors like monopolar energy usage, the size of the instrument, and the length of post-surgery catheterization are commonly linked to this outcome. Nonetheless, the reasons and risk elements leading to stricture formation are still widely discussed. Other considered contributors include slow resection speed, extended surgical duration, reduced prostatic size, temperature of the irrigation solution, and the size and repetition of transurethral instrument passage [141–145].

### Late postoperative complications: bladder neck stenosis

**Fig. 16:**
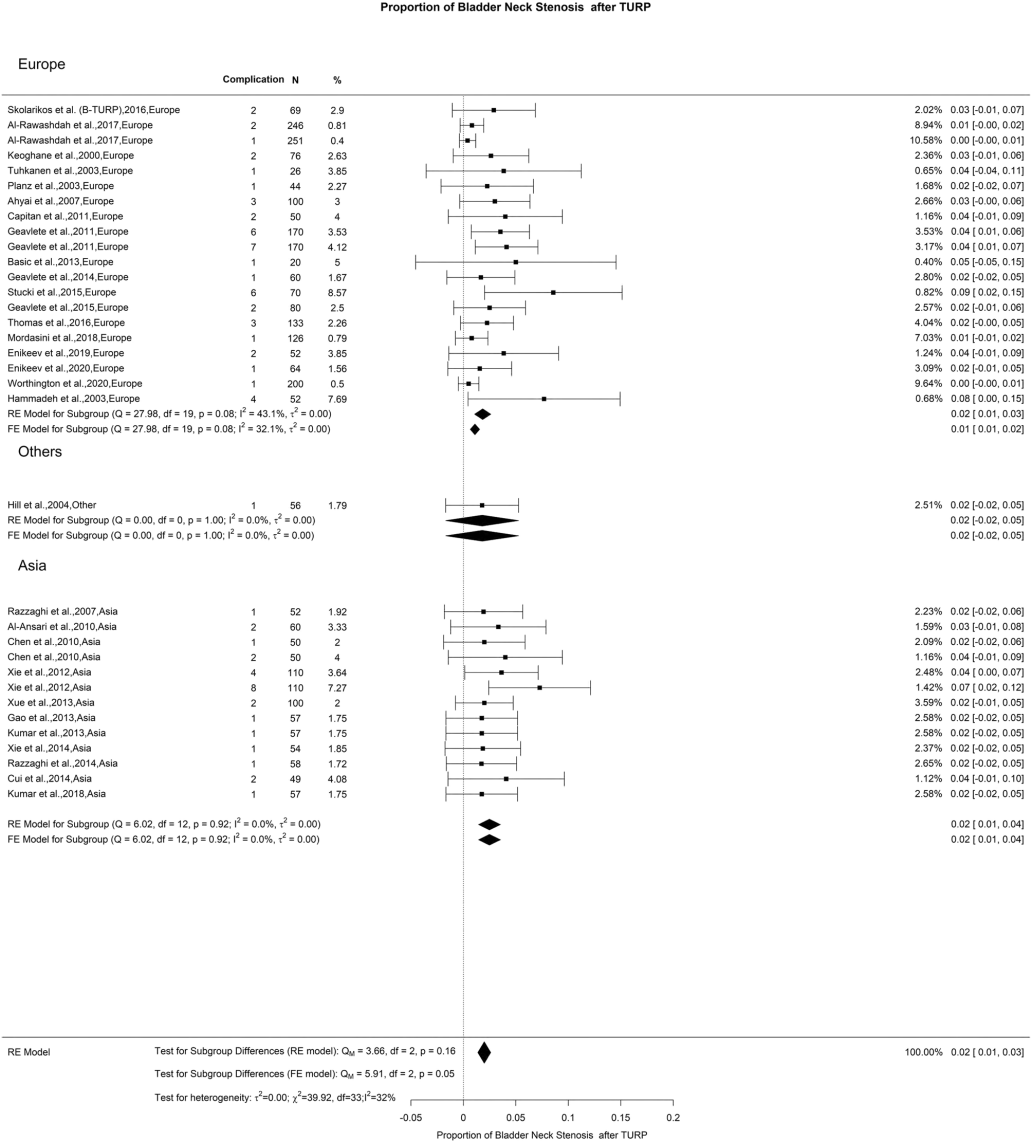
Proportion of Bladder Neck Stenosis after TURP

We observed a 2% BNS rate following TURP, accompanied by a moderate heterogeneity (I^2^ = 31%). There were no notable discrepancies (Fig. 16). Previous studies have established a correlation between BNS and prostate sizes under 30 g, presenting incidence rates from 0.3 to 9.2% [133, 146, 147]. While the incidence of BNS appears consistent after TURP, enucleation, and ablative methods, it is essential to highlight that the results of endoscopic interventions for BNS may vary considerably based on the initial procedure causing the issue. Success rates for BNS treatments appear to be greater after endoscopic enucleation procedures (EEP) as opposed to TURP [148, 149].

### Incidental prostate cancer

**Fig. 17:**
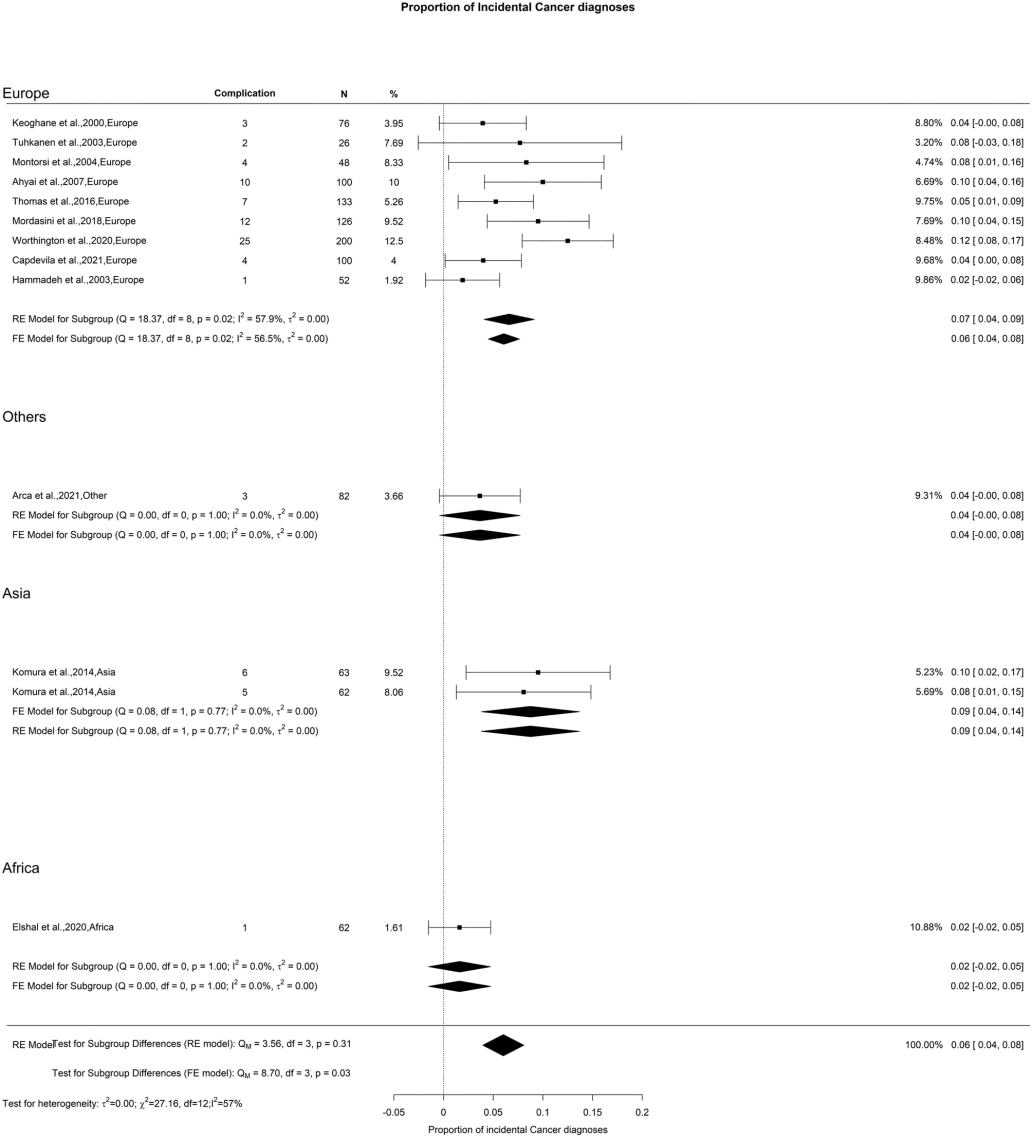
Proportion of Incidental Prostate Cancer after TURP

The total diagnosis rate of iPCa post-TURP was 6%, accompanied by a moderate OH (I^2^ = 61%). The analysis did not reveal any significant discrepancies (Fig. 17). Nevertheless, as highlighted by other researchers, the occurrence of iPCa is lower with TURP than with EEP [150]. This could be due to the more complete tissue removal achieved by EEP compared to TURP.

### Reduction in prostate volume, PSA, and retreatment rate

**Fig. 18:**
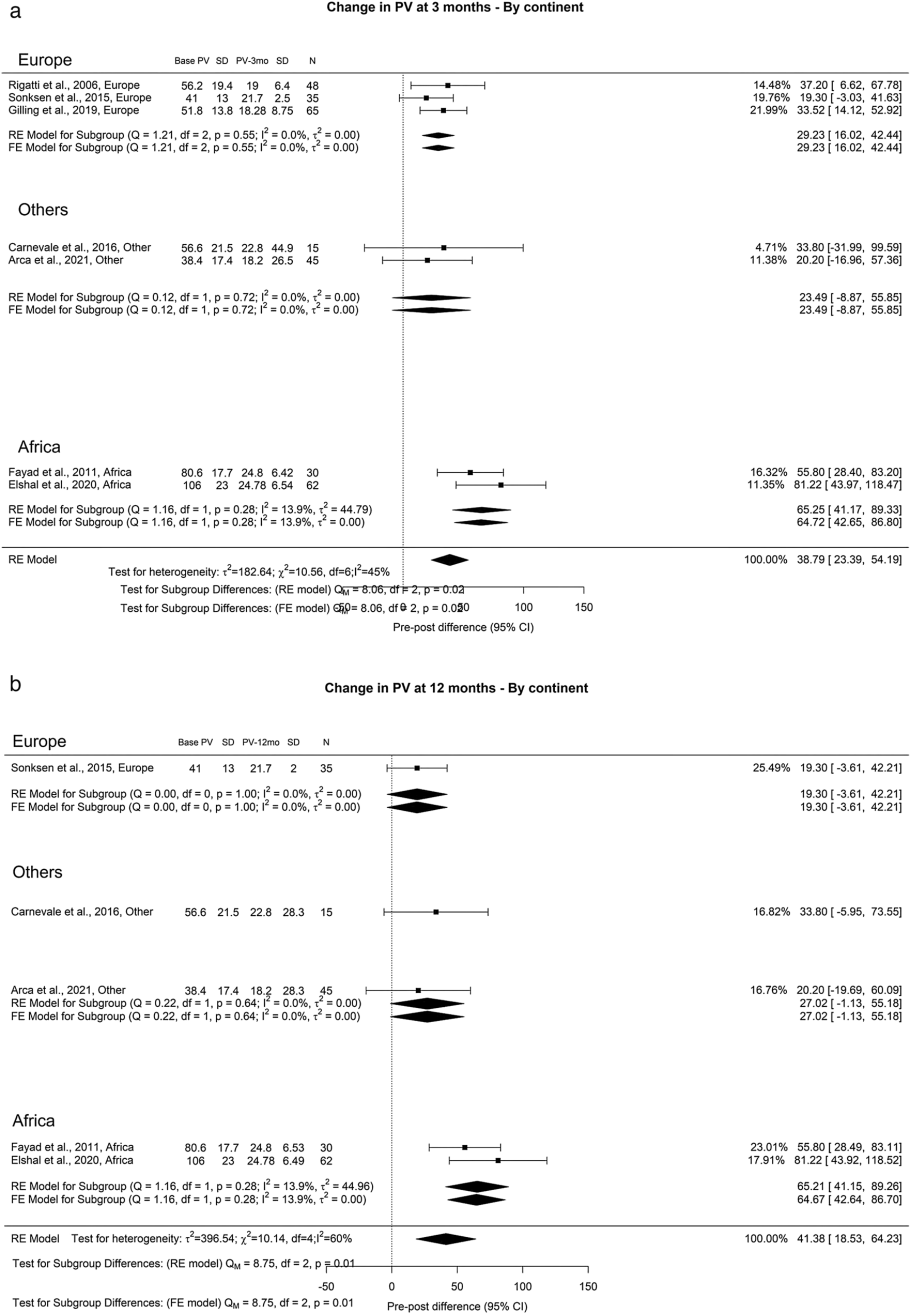
Change in Prostate Volume by continent. **A**. At 3 months; **B**. At 12 months

Three months post-procedure, the remaining prostate volume was 38.79 g, which increased slightly to 41.38 g at the 12-month mark. Noteworthy differences were identified in the regional analysis. After 3 months (Qm = 8.06, *p* = 0.02), the prostate volume disparities in Europe, Other regions, and Africa stood at 29.23, 23.49, and 65.25 g, respectively. At the 12-month point (Qm = 8.75, *p* = 0.01), the reductions in prostate volume were 19.30, 27.02, and 65.21 g for Europe, Other regions, and Africa, respectively (Fig. 18).

**Fig. 19:**
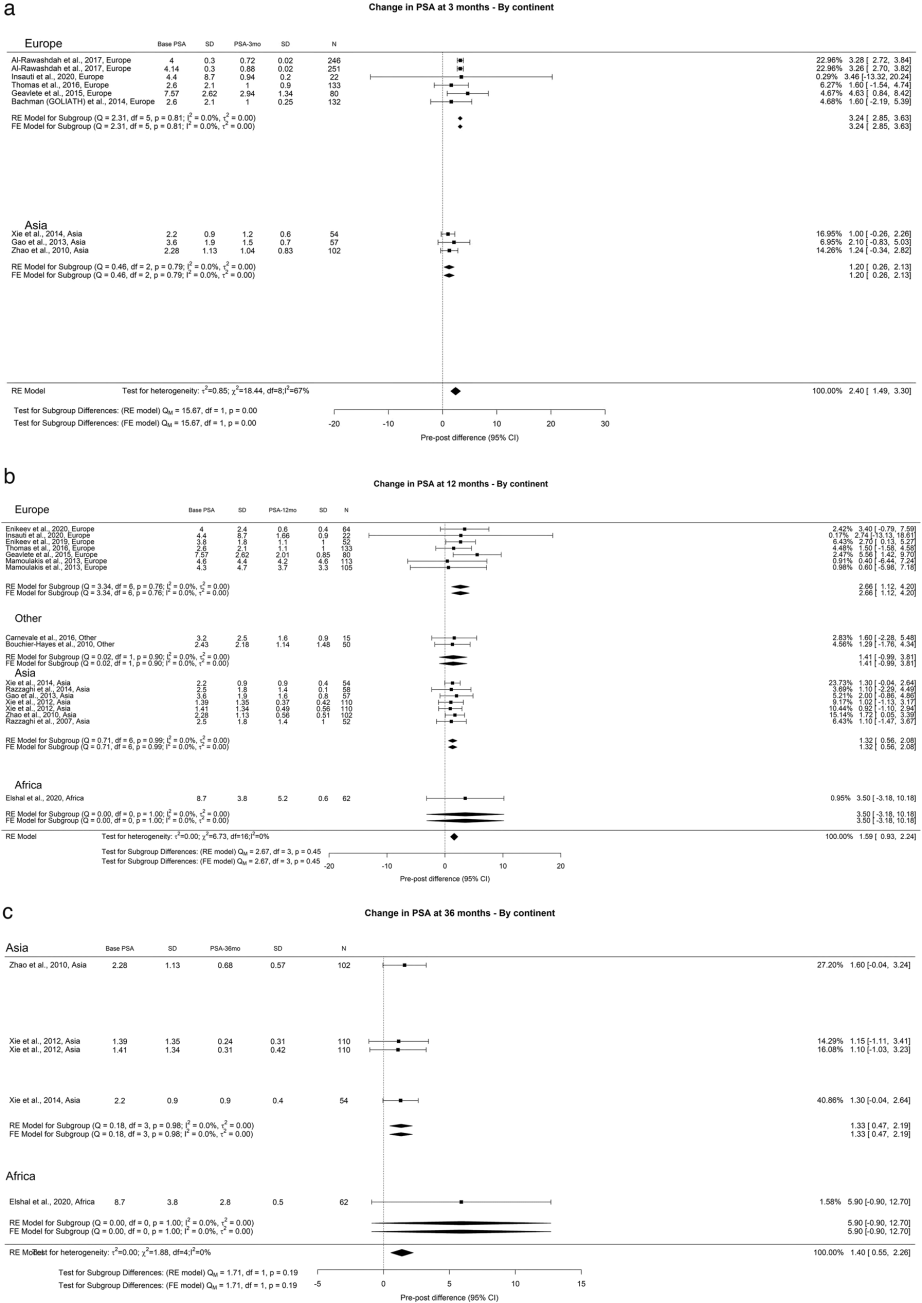
Change in PSA by continent. **A**. At 3 months; **B**. At 12 months; **C**. At 36 months

The aggregate PSA reduction at 3-month, 12-month, and 36-month were 2.40 ng/ml, 1.59 ng/ml, and 1.40 ng/ml, respectively. After 3 months (Qm = 15.67, *p* = 0.00, I^2^ = 67%), Europe and Asia witnessed PSA decreases of 3.24 ng/ml and 1.2 ng/ml, respectively. By the 3-year mark, Asia recorded a PSA of 1.33 ng/ml, while Africa saw a notable 5.90 ng/ml (Fig. 19).

**Fig. 20:**
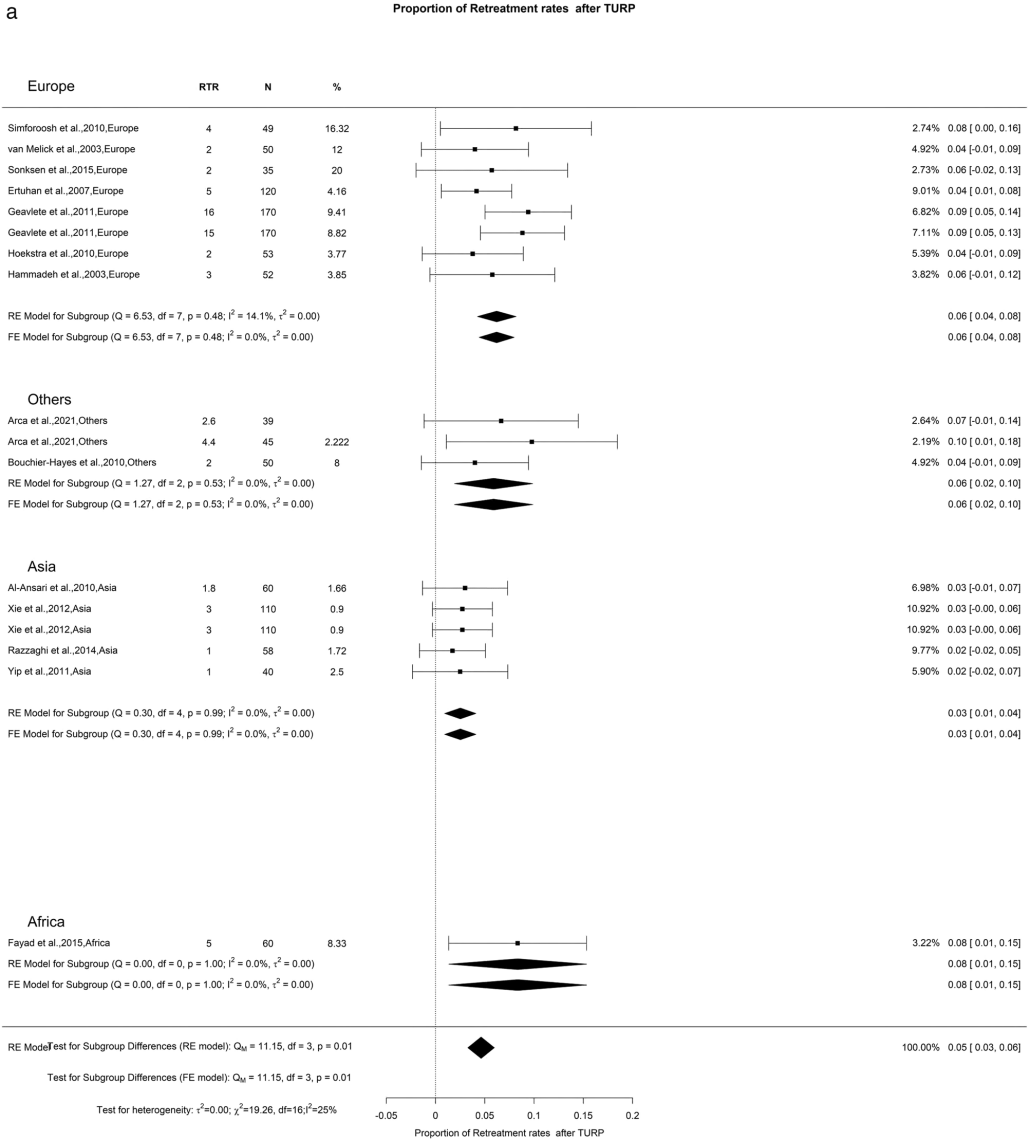

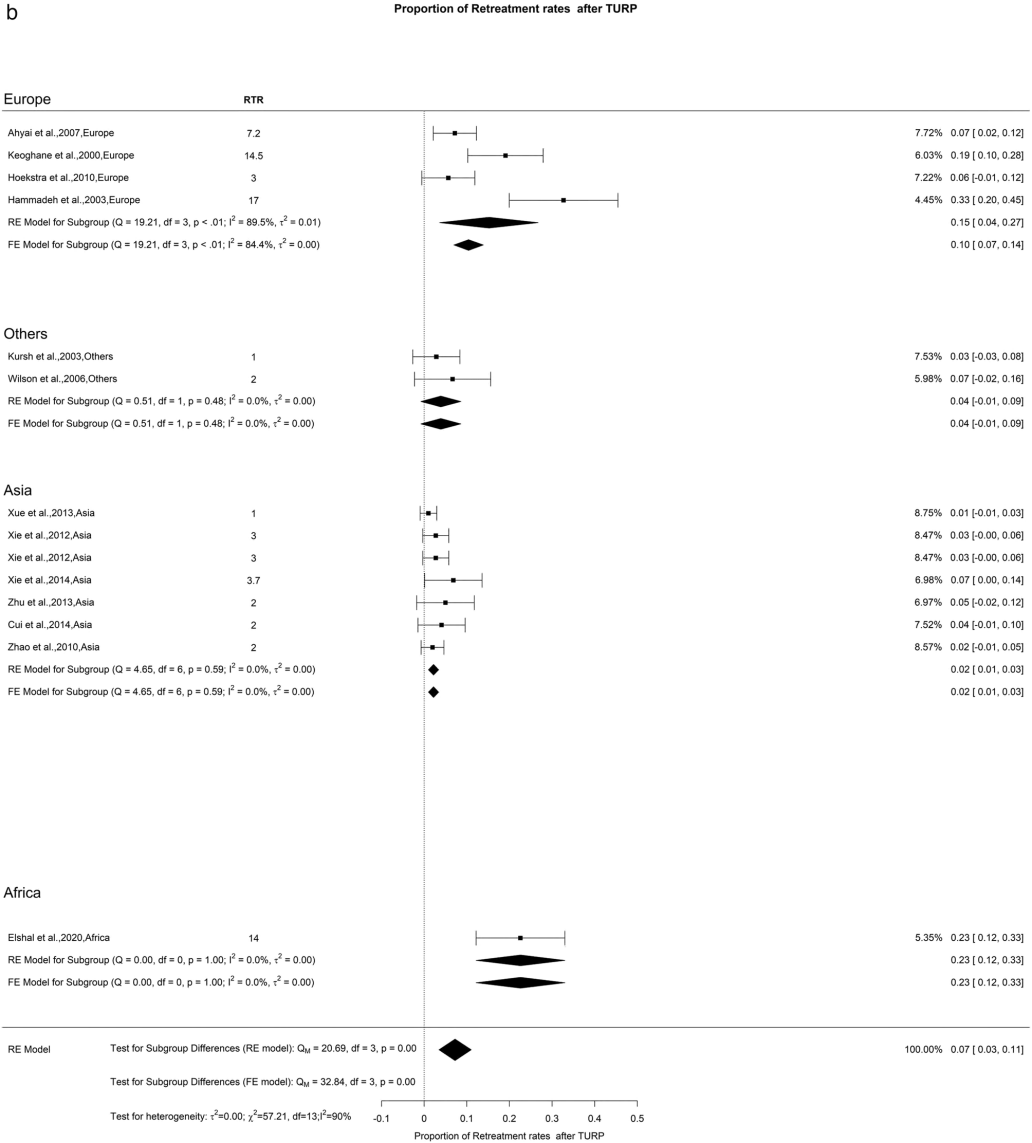
Proportion of Retreatment after TURP. **A**. At 12 months; **B**. At 36 months

In our research, we examined the retreatment rate at both 12- and 36-month post-procedure. At the 12-month interval, the rate stood at 5% with a low level of OH (I^2^ = 25%). A significant difference was seen with Europe, Others, Asia, and Africa showing retreatment rate at 1 year of 6%, 6%, 3%, and 8%, respectively. Furthermore, we found a retreatment rate of 7% at 3 years, though the OH was notably higher (I^2^ = 90%). A deeper look into the 3-year data showed noteworthy regional differences (Qm = 10.72, *p* = 0.01). Specifically, Europe had a rate of 10%, other regions had 6%, Asia reported 5%, and Africa was 23% (Fig. 20). It is difficult to explain this difference in retreatment rate amongst different continents. We hypothesize that it might be related to difference in preoperative prostate sizes and amount of tissue resected during initial TURP.

Historically, there has not been a clear-cut definition regarding the ideal tissue volume to remove during TURP. This lack of a uniform benchmark for determining surgical thoroughness can lead to considerable variations in the volume of tissue ultimately resected [151, 152]. Such inconsistencies might clarify the disparities seen in regional analyses. A pivotal study from Africa by Elshal et al., which compared TURP techniques with Greenlight and holmium laser enucleation for prostates weighing between 80 and 150 g, was a primary determinant of improved outcomes in that region [37]. Our findings illustrate that the prostate gland experiences gradual growth over time, with factors like age, testosterone, and inflammatory markers being pivotal influencers [153]. Additionally, the correlation between prostate volume and PSA levels is well-documented, with general guidelines suggesting a PSA reduction of 0.1–0.3 ng/ml for every gram of tissue removed [152, 154–157]. Bhat et al. highlighted those various elements, such as surgeon expertise, prostate dimensions, age, and patient health conditions influence the PSA reduction after TURP. Among these, the resected prostate volume seems to be the most influential [152]. This study concurs with these findings, showing comparable patterns concerning prostate size and PSA.

According to research conducted by Frendl et al., instances where patients faced acute urinary retention or needed retreatment for LUTS were deemed as treatment failures post-TURP. Over a span of seven years, this study documented a failure rate of 15.27% [9].

## Strengths and limitations

Our research has several constraints worth mentioning. First, diversity in baseline characteristics, along with insufficient reporting on key patient comorbidities such as associated neurologic conditions, underactive bladder, diabetes mellitus, concomitant medications may limit the broader applicability of our conclusions. Although urodynamic studies are not mandatory before TURP, the absence of such data limits the ability to identify coexistent underactive detrusor, which may negatively impact TURP outcomes [158]. The short-term monitoring in many studies may not capture the full trajectory of TURP outcomes. Variability in surgical methods and aftercare procedures across studies could also skew our analysis. The inconsistent inclusion criteria in clinical trials, with roughly 40% omitting patients without a precise definition, create challenges in assessing treatment results, such as retreatment rates [122, 159]. We also did not distinguish between TURP variants like monopolar, bipolar, and plasmakinetic, which could affect efficacy and complication rates. Different TURP methods might have diverse complication profiles, including TURP syndrome and bleeding. We also did not separately report outcome of antegrade ejaculation sparing TURP. However, given the lack of substantial efficacy differences among these subtypes, we aimed to address TURP holistically. Evaluating surgical measures in both monopolar and bipolar TURP in terms of effectiveness is a sensible tactic. Also, most RCTs took place in prominent tertiary care institutions, which might not accurately depict the wider context of TURP practices and outcomes.

Despite these constraints, our research provides a detailed overview of TURP outcomes worldwide, highlighting areas that warrant more detailed investigation to optimize patient care. For instance, the noticeable heterogeneity in outcomes like bleeding, clot removal, incontinence, irritative symptoms, and UTIs indicates the need for more uniform outcome reporting in TURP clinical trials. A key strength of our research is its extensive analysis, offering an in-depth perspective on TURP outcomes. By shedding light on varying outcomes, our study lays a foundation for future research aiming to enhance TURP's safety and effectiveness.

## Future perspectives for research

This meta-analysis underscores the notable inconsistency in how outcomes are reported across studies, especially regarding complications like bleeding and irritative symptoms. To rectify this, it is crucial for upcoming clinical trials to embrace uniform outcome metrics and reporting standards, thereby improving study comparability and fostering stronger analyses. Formulating a universally agreed-upon set of core outcomes would markedly augment the credibility and consistency of subsequent research, guaranteeing more precise inter-study comparisons.

As technological advancements continue to emerge, novel surgical methods, including various laser techniques, are being introduced as potential alternatives [160–162]. Although many studies have been published on various types of laser prostatectomy, a real worldwide comparison between lasers and TURP would be challenging. This meta-analysis underscores inconsistent outcome reports across TURP-related studies, especially regarding side effects. Such irregularities can undermine confidence in the results of studies examining new methods since the conventional reference point is not consistently depicted. This becomes especially pertinent considering the rise of MISTs, which tout comparable efficacy and reduced complications relative to TURP. As a result, there is an imperative to conduct comparative research comparing the results of these nascent techniques with TURP, facilitating the identification of the most effective and minimally invasive treatments.

Additionally, it is important to point out that previous studies have shown that most clinical trials globally are conducted in Europe, which may explain the predominance of European and Asian studies in BPH research [163]. Given that BPH studies usually do not rely on external funds, this trend likely reflects stronger academic incentives in these regions for conducting such studies, compared to North America, where research is more often driven by sponsor-supported projects. Future studies should investigate the impact of funding models and regional academic priorities on the geographic distribution of BPH research.

Moreover, BPH significantly influences both the well-being of patients and healthcare expenditures. Upcoming research endeavors should integrate evaluations of patients' quality of life and undertake cost-effectiveness analyses. By encompassing these dimensions, a holistic appraisal of these innovative treatments can be achieved, aiding informed choices and ensuring the best use of medical resources.

## Conclusions

This research indicates that global outcomes for TURP remain inconsistent. Our findings suggest that while subjective outcome measures, based on self-reports, are fairly consistent across regions, objective measures exhibit significant differences. PVR and Qmax showed the most pronounced variation. Such intracontinental variability might point to a lack of standardization in TURP procedures probably related to differences in surgical techniques and template, surgeon expertise, patient characteristics including baseline prostate volume. Yet, the consistent improvement in symptoms across patients suggests that, regardless of regional differences in procedure execution, TURP remains the premier choice for BPH treatment.

## Supplementary Information

Below is the link to the electronic supplementary material.

Supplementary file1 (DOCX 4526 KB)

## Data Availability

No datasets were generated or analysed during the current study.
