# Supplementary material for: Transurethral resection of the prostate across continents: a meta-analysis evaluating quality of gold standard in the twenty-first century
Source: World J Urol. 2025 Jan 24;43(1):85. doi: 10.1007/s00345-024-05439-7 (PMC11761131; doi:10.1007/s00345-024-05439-7)
Supplement: Supplementary file 1 — Supplementary file1 (DOCX 4526 KB) [file 345_2024_5439_MOESM1_ESM.docx]

**Supplementary Figure 1. PRISMA 2020 flow diagram for systematic reviews**

**Identification of studies via databases and registers**

Records (from January 1^st^, 2000, to December 31^st^, 2022) identified through database searching **PubMed** (n = 551)

**Identification**

Records screened

(n = 551)

Records excluded based on title.

(n = 248)

**Screening**

Records screened

(n = 303)

Records excluded based on abstract (n = 159)

**Eligibility**

Full-text articles excluded

(n = 42)

Full-text articles assessed for eligibility

(n = 144)

Studies included in qualitative synthesis.

(n = 102)

**Included**
